# Gene co-expression networks from RNA sequencing of dairy cattle identifies genes and pathways affecting feed efficiency

**DOI:** 10.1186/s12859-018-2553-z

**Published:** 2018-12-17

**Authors:** S. M. Salleh, G. Mazzoni, P. Løvendahl, H. N. Kadarmideen

**Affiliations:** 1 Department of Veterinary and Animal Sciences, Faculty of Health and Medical Sciences, University of Copenhagen, DK-1870 Frederiksberg C, Denmark; 2 Department of Animal Science, Faculty of Agriculture, Universiti Putra Malaysia, UPM, 43400 Serdang, Selangor Malaysia; 3 Department of Bio and Health Informatics, Technical University of Denmark, DK-2800 Kgs. Lyngby, Denmark; 4 Department of Molecular Biology and Genetics - Center for Quantitative Genetics and Genomics, Aarhus University, AU Foulum, DK-8830 Tjele, Denmark; 5 Department of Applied Mathematics and Computer Science, Technical University of Denmark, DK-2800 Kgs. Lyngby, Denmark

**Keywords:** RNA-seq, Feed efficiency, Residual feed intake, Co-expressed genes, Hub genes, Pathways, Holstein, Jersey, Dairy cattle

## Abstract

**Background:**

Selection for feed efficiency is crucial for overall profitability and sustainability in dairy cattle production. Key regulator genes and genetic markers derived from co-expression networks underlying feed efficiency could be included in the genomic selection of the best cows. The present study identified co-expression networks associated with high and low feed efficiency and their regulator genes in Danish Holstein and Jersey cows.

RNA-sequencing data from Holstein and Jersey cows with high and low residual feed intake (RFI) and treated with two diets (low and high concentrate) were used. Approximately 26 million and 25 million pair reads were mapped to bovine reference genome for Jersey and Holstein breed, respectively. Subsequently, the gene count expressions data were analysed using a Weighted Gene Co-expression Network Analysis (WGCNA) approach. Functional enrichment analysis from Ingenuity® Pathway Analysis (IPA®), ClueGO application and STRING of these modules was performed to identify relevant biological pathways and regulatory genes.

**Results:**

WGCNA identified two groups of co-expressed genes (modules) significantly associated with RFI and one module significantly associated with diet. In Holstein cows, the salmon module with module trait relationship (MTR) = 0.7 and the top upstream regulators ATP7B were involved in cholesterol biosynthesis, steroid biosynthesis, lipid biosynthesis and fatty acid metabolism. The magenta module has been significantly associated (MTR = 0.51) with the treatment diet involved in the triglyceride homeostasis. In Jersey cows, the lightsteelblue1 (MTR = − 0.57) module controlled by IFNG and IL10RA was involved in the positive regulation of interferon-gamma production, lymphocyte differentiation, natural killer cell-mediated cytotoxicity and primary immunodeficiency.

**Conclusion:**

The present study provides new information on the biological functions in liver that are potentially involved in controlling feed efficiency. The hub genes and upstream regulators (ATP7b, IFNG and IL10RA) involved in these functions are potential candidate genes for the development of new biomarkers. However, the hub genes, upstream regulators and pathways involved in the co-expressed networks were different in both breeds. Hence, additional studies are required to investigate and confirm these findings prior to their use as candidate genes.

**Electronic supplementary material:**

The online version of this article (10.1186/s12859-018-2553-z) contains supplementary material, which is available to authorized users.

## Background

Globally, food demand is increasing as a consequence of world population growth [1]. However, arable land to produce sufficient amounts of food is decreasing, and the carbon footprint is increasing [2]. Hence, solutions for efficient and environmentally friendly methods to produce food are urgently needed.

Feed efficiency (FE) in dairy cattle is the ability of a cow to convert the feed nutrient consumed into milk and milk by-products. Many approaches have been developed and adopted to select the most feed-efficient cows. Currently, residual feed intake (RFI) has been used to measure FE in dairy cows [3, 4]. Residual feed intake is the difference between the predicted and actual feed intake [5]. Regression models have been used to calculate the RFI value. Thus, animals with low RFI values are more efficient [6]. The genetic selection of animals with a low RFI will improve profitability [7], decrease greenhouse gasses emissions [8] and optimize the use of food resources. However, in the case of dairy cattle, the interpretation of RFI is not straightforward. Many other factors should be considered, as this selection might lead to a negative energy balance, cause health issues and affect the fertility of the cows [9, 10].

In Denmark, Holstein and Jersey are the most common dairy breeds used [11]. Comparatively, Holstein and Jersey cattle do not differ in terms of digestibility, energy efficiencies, and the ability to convert dietary protein to milk protein [12]. However, there are no gene expression profiling studies of these breeds. Hence, to understand the complex biological mechanisms in nutrient partitioning in dairy cattle, liver transcriptomics analysis may be useful to interpret and understand the pathways and functional elements of the genomes involved [13]. Transcriptomics is a form of high throughput analysis to quantify gene expression in a specific cell type or tissue [14]. Various studies have reported that mRNA levels of many genes are heritable, which affects genetic analysis [15–17]. Many studies based on transcriptomics (microarray and RNA-sequencing) have been conducted to study gene expression in feed efficiency [18–20]. Studies on differential gene expression have been well established to identify candidate genes for biomarker development [21]. There are limited studies related to gene expression for RFI traits in dairy cattle, particularly for Jersey and Holstein breeds. However, some studies have reported the gene expression associated with RFI in other breeds and species. For example, Lkhagvadorj et al. [22] found that the common energy consumption controlled by *PPARA*, *PPARG* and/or *CREB* is related to RFI in pigs. In beef cattle, Alexandre et al. [19] reported the alteration of lipid metabolism and an increase in the inflammatory response in animals with low feed efficiency. Paradis et al. [20] also reported a greater response to hepatic inflammation in heifers with high feed efficiency. In Nellore beef cattle, Tizioto et al. [23] identified the differentially expressed genes involved in oxidative stress. Hence, transcriptomics analysis might provide additional knowledge on the complex mechanisms that regulate nutrient intake.

Diet affects the energy metabolism and efficiency of dairy cows [24]. Some studies have investigated the correlation between FE and diet, focusing on the gene expression profiles of specific tissues. Dairy cows are typically fed high energy or high-concentrate feed to meet the high-energy demand during the lactation period. It has previously been shown that high energy feeding does not affect the fatty acid concentration but does affect the expression of genes such as *ACACA*, *LPL* and *SCD* in the lipid metabolism [25]. Thus, it is also interesting to investigate the effects of different levels of energy in feed using co-expression network approaches.

Previously, we performed differential gene expression analysis on RNA from the livers of Holstein and Jersey cows. We identified several differentially expressed genes between high and low RFI [26]. The differentially expressed genes were related to primary immunodeficiency, steroid hormone biosynthesis, retinol metabolism, starch and sucrose metabolism, ether lipid metabolism, arachidonic metabolism and cytochrome P450 in drug metabolism. These biological processes and pathways are important mechanisms that are associated with feed efficiency.

Therefore, it is important to thoroughly investigate the mechanisms controlling feed efficiency. Systems biology is the most promising approach to obtain a better understanding of complex traits, such as feed efficiency. In systems biology, many computational methods are based on network approaches. Co-expression network analysis has been successfully used to analyse complex traits and diseases in humans and animals [27–30]. Weighted Gene Co-expression Network Analysis (WGCNA) can be used to identify clusters (modules) of highly correlated genes [31]. WGCNA has been used to identify candidate genes that are associated with the FE. Alexandra et al. (2015) identified differentially co-expressed genes that are involved in lipid metabolism in RFI divergent Nellore cattle. Similarly, lipid metabolism-related processes were identified in low-RFI pigs [22].

In the present study, the WGCNA method was applied to RNA-Seq data from the livers of Holstein and Jersey cows to: i) identify groups of co-expressed genes and biological pathways associated with RFI; ii) identify the hub genes and upstream regulators in these modules that may be good candidate genes for feed efficiency-related traits; and iii) compare the mechanisms and processes involved in RFI between Holstein and Jersey cattle. To our knowledge, this study is the first to use weighted gene network approaches to examine the overall complex transcriptional regulation of feed efficiency (RFI) using RNA-Seq data in Danish Holstein and Jersey cows.

## Materials and methods

### Animal ethics statement

The experimental design and animals that were being used in this experiment were permitted by the Danish Animal Experimentation Inspectorate.

### Experimental data

The experimental design and details of the experimental animals have been previously described in [26].

In brief, the dataset used in this experiment consists of 38 RNA-Seq expression profiles of liver bioposies from nine Holsteins and ten Jersey cows. In each breed group, cows were classified in high and low feed efficient and RNA samples were collected before and after treatment diet (low and high concentrate diet). The animals were assigned to the different diets after at least for 14–26 days adaptation period. All 38 RNA samples were paired-end sequenced using Illumina HiSeq 2500. The bioinformatics pipeline for RNA-Seq data processing is described in [26]. The expression quantification was performed using *Ensembl* Bovine annotation (release 82). The raw count data matrix used in this study is available in http://www.ncbi.nlm.nih.gov/geo/query/acc.cgi?acc=GSE92398.

### Weighted gene co-expression network analysis (WGCNA)

The Weighted Gene Co-expression Network Analysis (WGCNA) [31] R package was used to build co-expression networks and identify groups of highly co-expressed genes. Individual analyses were conducted on each breed group.

First, the low count genes and outliers were filtered by leaving only genes that had at least 1 count per million in 90% of the group. The remaining 11,153 genes in Holstein and 11,238 genes in Jersey were used for the analysis. The gene expression counts were normalized using the default procedure from the DESeq2 package version 1.12.0 [32] by correcting for the parity number to reduce potential effects from the parity number factor. The normalized data were subsequently log transformed as suggested in the WGCNA manual (https://horvath.genetics.ucla.edu/html/CoexpressionNetwork/Rpackages/WGCNA/). The final dataset was used in WGCNA to build an unsigned network. Pairwise Pearson’s correlations among all genes were calculated to create an adjacency matrix. A soft threshold power was set at β = 12 for Holstein and β = 10 for Jersey, correspondent to a scale-free topology index (R^2^) [33] of 0.9 for Holstein and 0.8 for Jersey. The adjacency matrix was used to calculate the Topological Overlap Measure (TOM). Modules of co-expressed genes were identified by using the dynamic tree cut algorithm [34]. Modules were arbitrarily labelled with different colours.

The module eigengenes were computed for each module using the first principal component to capture the variation in gene expression within each module. The eigengene sign was chosen to have a positive correlation with average module gene expression.

The correlation between module eigengene and RFI or treatment diet was evaluated to select modules that were associated with the respective traits (*p*-value < 0.05). In addition, FDR were computed using Benjamini–Hochberg (BH) method separately for each breed.

Gene significance (GS) was computed for each gene as the correlation between gene expression counts and FE. In addition, hub genes were identified, selecting genes with high module membership (MM > 0.8) in the modules of interest.

### Functional enrichment analysis

The modules that are significantly associated with RFI and treatment diet traits were selected.

Functional enrichment analysis was performed in the selected modules to identify and interpret complex biological functions based on gene ontology terms for the biological processes, molecular functions and cellular components and based on the KEGG pathways annotation.

All the genes included in each module were used in the functional enrichment analysis with the Cytoscape 3.4.0 plug-in software, ClueGO v2.2.6 [35]. The significance value was set as *p*-value < 0.05 and the BH correction was used as the multiple test correction. The reference set used for this analysis included a total of 9064 genes. The list of genes in the module of interest was also analysed using the STRING v.10.0 [36] database and the *Bos taurus* annotation.

Ingenuity® Pathway Analysis (IPA®) was used to detect upstream regulators, diseases and functions in the selected modules. The upstream regulator analysis identifies the upstream regulators that better explain the change in gene expression. The analysis is based on the set of indirect relationships present in the IPA® database. The algorithm computes an overlap *P*-value by measuring enrichment of network-regulated genes to determine the most likely set of upstream regulators. Next, the algorithm computes the activation Z-score by identifying the match of up- and down-regulation annotated in Ingenuity knowledge base. The Z-score is then used to predict the activation state of the upstream regulators (either activated or inhibited).

**Fig. 1:**
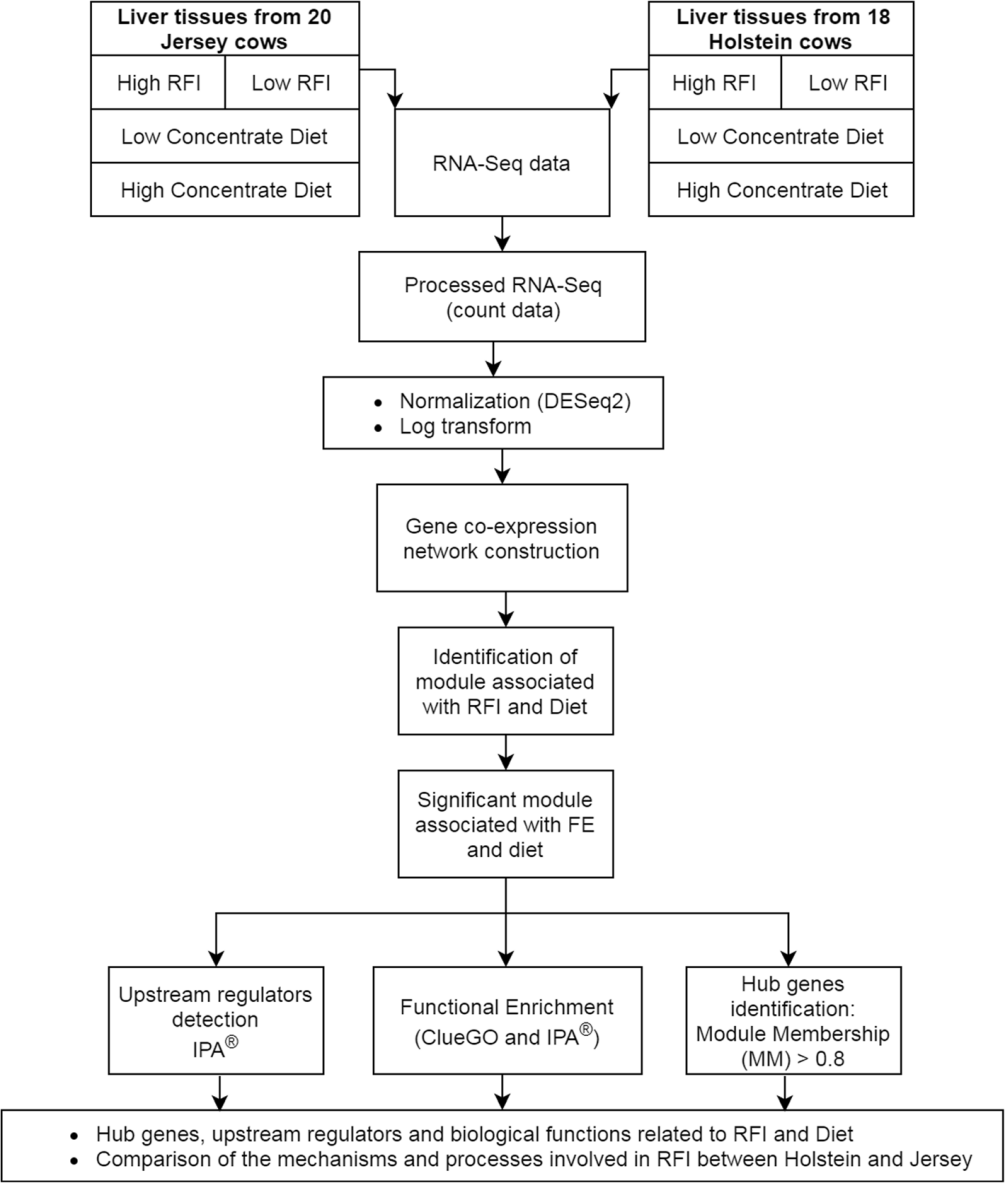
Experimental design and co-expressed gene network analysis pipeline

A summary of the pipeline of the experimental workflow, bioinformatics and statistical analysis is presented in Fig. 1.

## Results

**Fig. 2:**
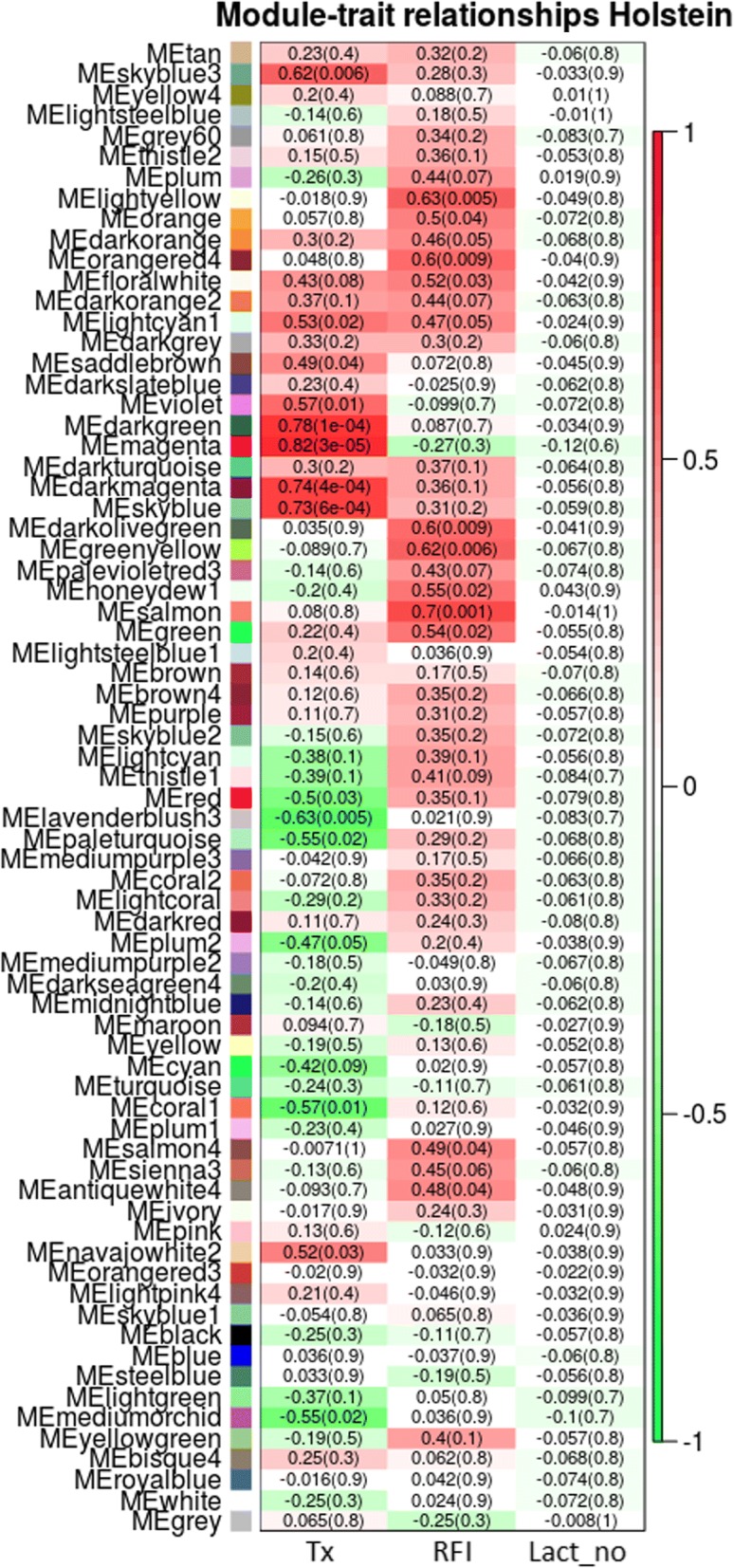
Module trait relationship (p-value) for detected modules (y-axis) in relation with traits (x-axis) for Holstein cows. The module trait relationship were colored based on the correlation between the module and traits (red = strong positive correlation; green = strong negative correlation). X-axis legend: Diet = Treatment diet; RFI = Residual feed intake; Lact_no = Lactation number

**Fig. 3:**
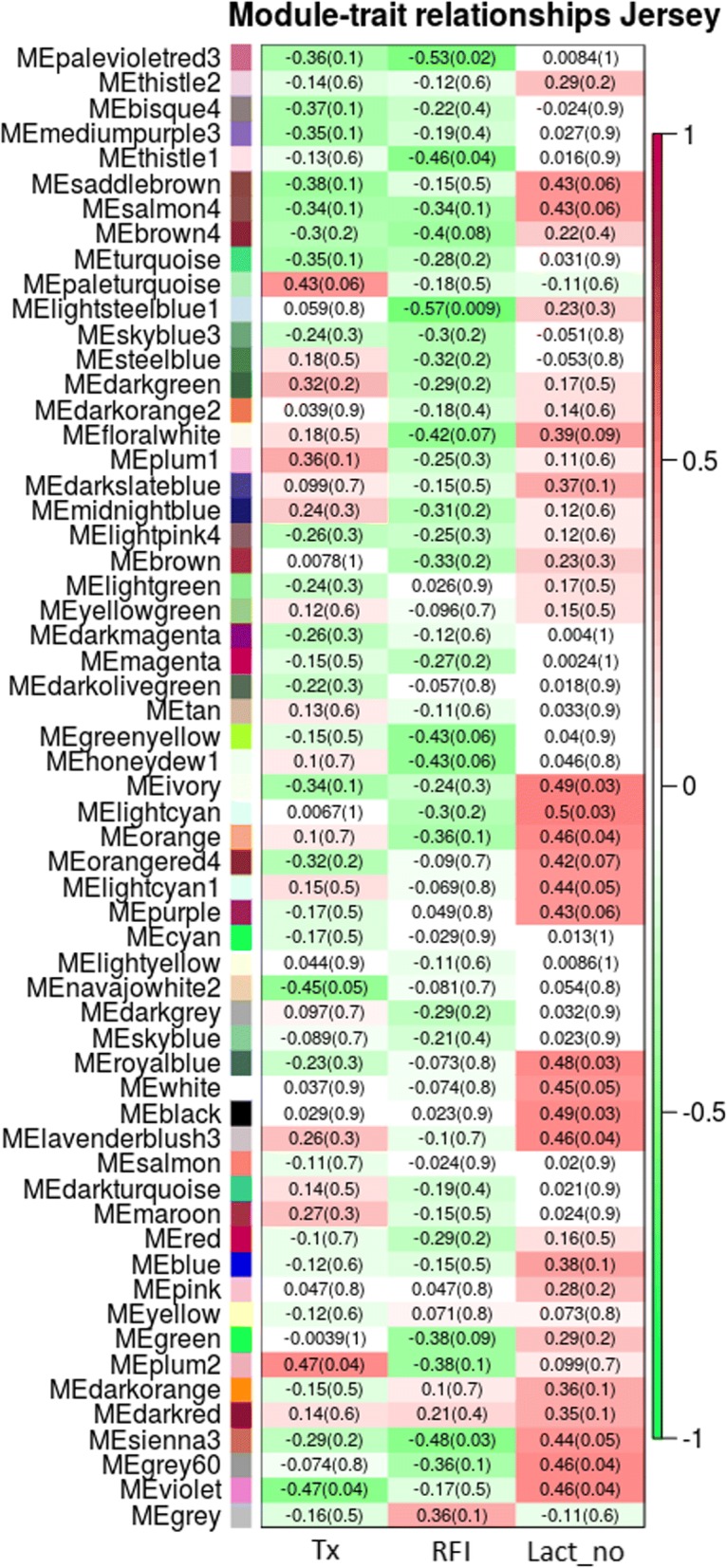
Module trait relationship (*p*-value) for detected modules (y-axis) in relation with traits (x-axis) for Jersey cows. The module trait relationship were colored based on the correlation between the module and traits (red = strong positive correlation; green = strong negative correlation). X-axis legend: Diet = Treatment diet; RFI = Residual feed intake; Lact_no = Lactation number

In the present study, WGCNA was used to identify RFI and diet-associated co-expression modules and their key functions. In total, 72 modules (Fig. 2) for Holstein cows and 59 modules (Fig. 3) for Jersey cows were identified. Subsequent the module detection, we have performed multiple testing corrections (Additional file 1: Tables S1 and S2 in each breed using BH method despite the norm that it is not carried out across gene network modules and traits. Unfortunately, after the multiple testing corrections, none of the top module is significant at adjusted *p*-value < 0.05 and therefore the results are to be validated in independent experiments with larger sample size, which is beyond the scope of this study. The results reported here are therefore are of exploratory and preliminary in nature. Therefore, modules with nominal p-value< 0.05 were used to be reported and discussed in the subsequent sections.

A total of 11 modules and four modules were significantly correlated with RFI for Holstein and Jersey cows, respectively. Additionally, 13 modules for Holstein and two modules for Jersey were significantly associated with treatment diet.

We assigned all the significant modules into the ClueGO application analysis to investigate the gene ontology (GO) and KEGG pathway-related functions with specific traits. The modules with the top significant module trait relationships (MTRs) were selected as the modules of interest in the present study. The modules lightsteelblue1 and violet in Jersey cows and the modules salmon and magenta in Holstein cows were selected for RFI and treatment diet, respectively.

### Modules related to RFI and treatment diet in Holstein cows

In Holstein cows, among the 11 modules that were significantly (*p*-value< 0.05) related to the RFI, salmon module (203 genes with MTR RFI = 0.7) is the top significant module. For the diet trait, we identified the magenta module as the top significant module. The magenta module comprised 212 genes that contribute to the MTR Diet = 0.82.

**Fig. 4:**
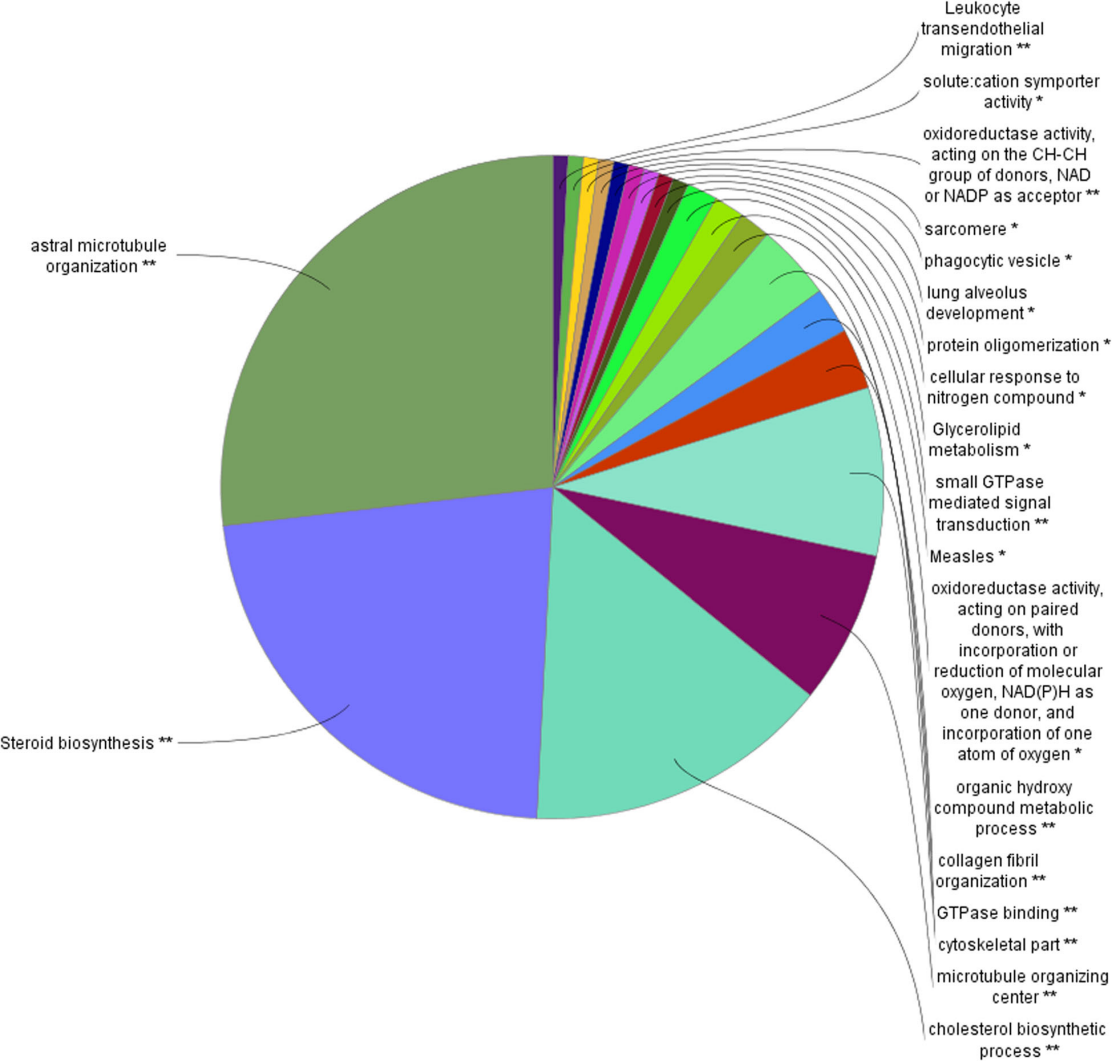
Pie chart presenting an overview of the significant GO terms and KEGG pathways in the salmon module in Holstein cows

In the top module (salmon), steroid biosynthesis was identified as the most enriched KEGG pathway (Fig. 4). This finding was also confirmed after analysing the genes in this module using www.string-db.org, and almost the same pathways and same patterns appeared in the output. Interestingly, most of the enriched pathways of co-expressed genes in Holstein cows were involved in steroid, lipid and cholesterol biosynthesis and metabolism (Fig. 4).

Additional file 1: Table S3 shows a summary of the functional groups with the number of genes involved in the GO terms and pathways. In total, 84 GO terms were significantly enriched (*p*-value< 0.05) after multiple testing corrections using BH. The GO-terms and KEGG pathways presented here are also almost the same as the output from the STRING 10 analysis (Additional file 1: Tables S5, S6 and S7).

The list of upstream regulators identified for the modules that are significantly associated with RFI and diet are presented in Additional file 1: Table S11. In the salmon module, ATP7B was predicted as activated, while POR and cholesterol were predicted as inhibited. In Additional file 1: Tables S13 and S14 shows the diseases and functions involved in salmon and magenta modules.

**Fig. 5:**
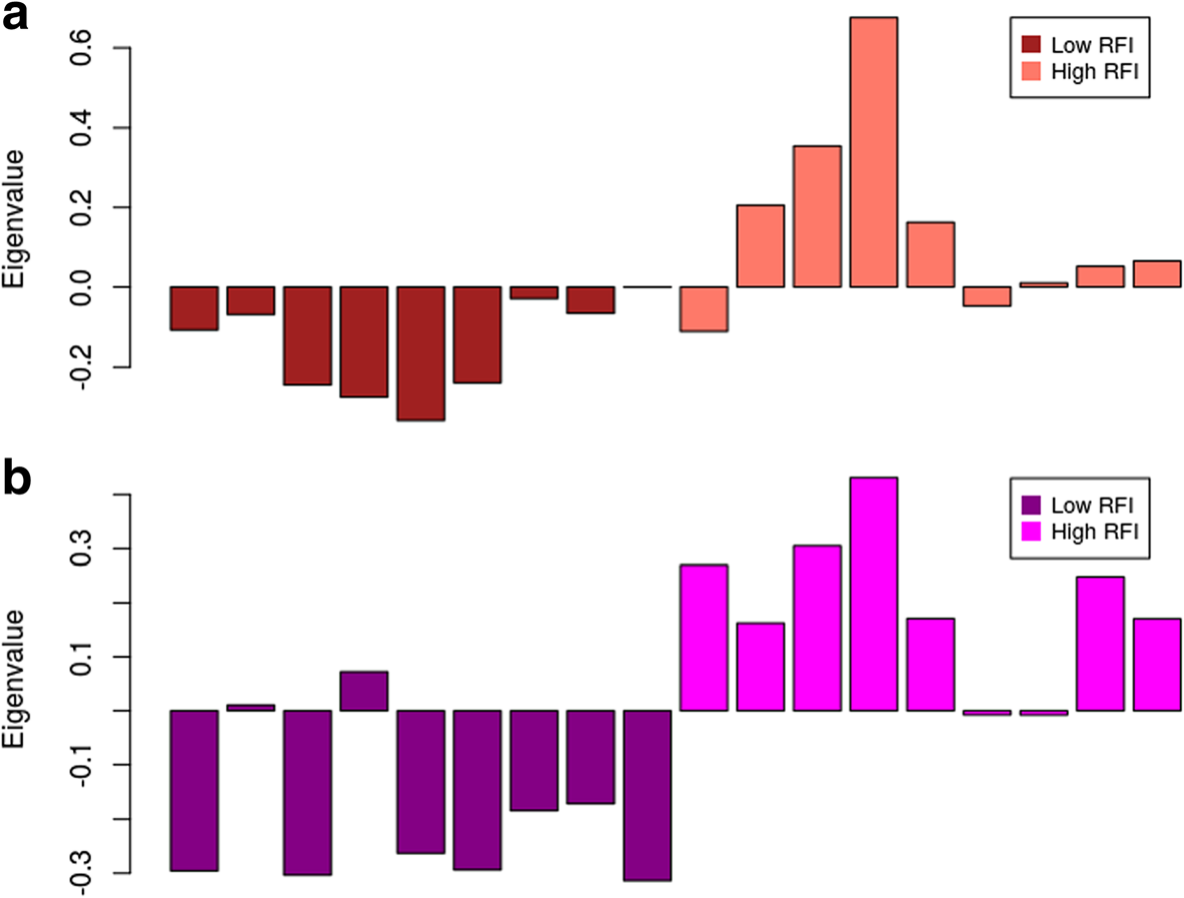
**a** Module eigengene (y-axis) across samples (x-axis) from the salmon module (associated to RFI) (**b**) Module eigengene (y-axis) across samples (x-axis) from the magenta module (associated to treatment diet)

The module eigengene diagram for both of the salmon and magenta modules shows a higher average expression profile in high RFI samples (Fig. 5 a and b).

The list of genes with high (MM > 0.8) in the salmon module is presented in Table 1.

**Table 1:** List of the top hub genes generated from (MM > 0.8) in the salmon module in Holstein cows

Ensembl gene ID	Gene name	Module membership	Gene significance
ENSBTAG00000000197	*TRMT10A*	0.801	0.576
ENSBTAG00000001774	*SPRY2*	−0.814	− 0.520
ENSBTAG00000001950	*RDH11*	0.852	0.441
ENSBTAG00000002412	*CYB5B*	0.907	0.633
ENSBTAG00000002435	*PTPRE*	0.852	0.767
ENSBTAG00000002714	*GNAI1*	0.901	0.557
ENSBTAG00000002827	*ACAT2*	0.946	0.691
ENSBTAG00000002966	*DNAJC13*	0.813	0.710
ENSBTAG00000003068	*MSMO1*	0.852	0.579
ENSBTAG00000003305	*NCF1*	0.802	0.642
ENSBTAG00000003696	*CCDC64*	0.837	0.679
ENSBTAG00000003718	*HACL1*	0.854	0.705
ENSBTAG00000003948		0.919	0.559
ENSBTAG00000004075	*IDI1*	0.870	0.607
ENSBTAG00000004688	*DHCR24*	0.859	0.555
ENSBTAG00000005183	*MVK*	0.906	0.497
ENSBTAG00000005498	*SQLE*	0.816	0.442
ENSBTAG00000005650	*SKAP2*	0.826	0.589
ENSBTAG00000005976	*HSD17B7*	0.809	0.550
ENSBTAG00000006999	*RYR1*	0.929	0.763
ENSBTAG00000007014	*CEP63*	0.823	0.623
ENSBTAG00000007079	*LCP1*	0.806	0.583
ENSBTAG00000007840	*HMGCR*	0.888	0.522
ENSBTAG00000007844	*CETN2*	0.836	0.335
ENSBTAG00000008160	*MBOAT2*	0.865	0.534
ENSBTAG00000008329	*CYTIP*	0.823	0.477
ENSBTAG00000010347	*EZR*	0.850	0.506
ENSBTAG00000011146	*RAB8B*	0.884	0.473
ENSBTAG00000011839	*HMGCS1*	0.871	0.507
ENSBTAG00000012059	*MVD*	0.831	0.364
ENSBTAG00000012170	*UBL3*	0.813	0.729
ENSBTAG00000012432	*FDFT1*	0.821	0.529
ENSBTAG00000012695	*LCK*	0.837	0.534
ENSBTAG00000013284		0.886	0.736
ENSBTAG00000013303	*ACSS2*	0.866	0.571
ENSBTAG00000013749	*RHOQ*	0.868	0.525
ENSBTAG00000014517	*KLB*	0.857	0.640
ENSBTAG00000015327	*SPTAN1*	0.899	0.637
ENSBTAG00000015980	*FASN*	0.859	0.490
ENSBTAG00000016445	*YME1L1*	0.807	0.717
ENSBTAG00000016465	*DHCR7*	0.903	0.521
ENSBTAG00000016709	*NT5C3A*	0.824	0.615
ENSBTAG00000016721	*ZNF791*	0.824	0.559
ENSBTAG00000016740	*ACLY*	0.918	0.520
ENSBTAG00000018936	*LSS*	0.839	0.580
ENSBTAG00000018959	*RAB11A*	0.828	0.670
ENSBTAG00000020984	*RAPGEF4*	0.856	0.775
ENSBTAG00000021842		0.804	0.492
ENSBTAG00000030951		0.844	0.508
ENSBTAG00000036260	*LPXN*	0.801	0.391
ENSBTAG00000037413	*TMEM164*	0.810	0.468
ENSBTAG00000047970		0.835	0.558

### Modules related to RFI and treatment diet in Jersey cows

Among the four modules significantly (*p*-value< 0.05) related to RFI in the Jersey group, the lightsteelblue1 module (72 genes) with a module trait relationship (MTR RFI = − 0.57) is the top significant (p-value< 0.05) module associated with RFI. In total, 44 GO terms were significantly enriched (*p*-value< 0.05) after multiple test correction using BH. For the diet trait, among the two significantly correlated modules, the violet module was the top significant (MTR Diet = − 0.47). However, this module has limited output from a functional enrichment analysis or no interesting biological information related to diet. Hence, the modules related to diet for the Jersey breed were not further discussed.

**Fig. 6:**
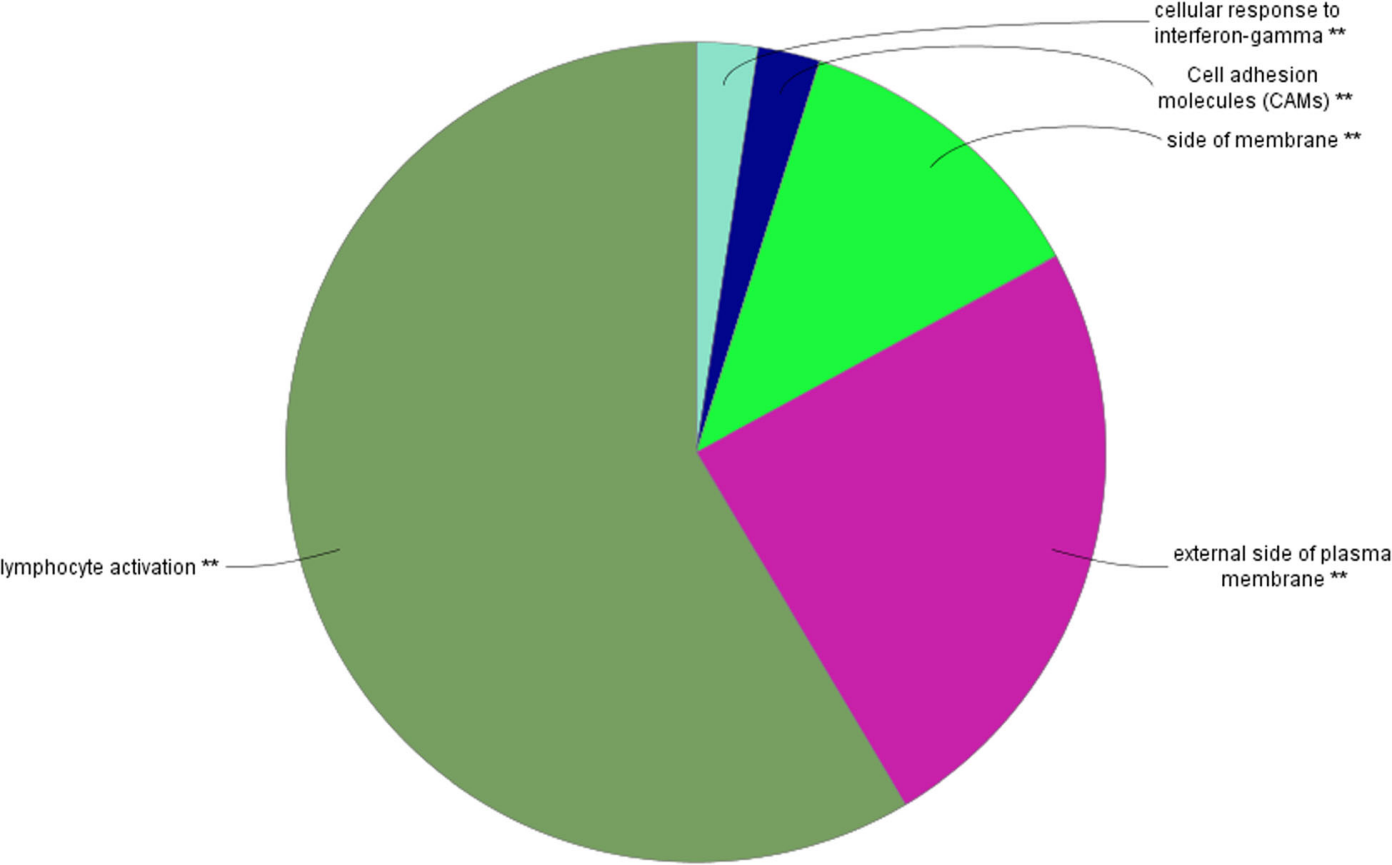
Pie chart visualization of GO terms and KEGG pathways in the lightsteelblue1 module in Jersey cows

Figure 6 and Additional file 1: Table S4 shows the top summarized GO terms involved in the lightsteelblue1 module that is related to immune system functions. The first and the second GO terms, which are associated with the regulation of lymphocyte activation and positive regulation of leukocyte activation, involved almost the same genes as those that are involved in immune system functions. In detail, primary immunodeficiency has been identified (p-value< 0.05) as a significant KEGG pathway that involves four genes together with the positive regulation of leukocyte activated GO terms.

We identified IFNG (Interferon Gamma) as inhibited and IL10RA (Interleukin 10 Receptor Subunit Alpha), NKX2–3 (NK2 Homeobox 3) and dexamethasone were predicted as activated upstream regulators (Additional file 1: Table S12). In Additional file 1: Tables S14 and S16 shows the diseases and functions involved in lightsteelblue1 and violet modules.

Interestingly, all of these upstream regulators have functions related to the immune system. In addition, GO-terms and KEGG pathways from the STRING 10 analysis (Additional file 1: Tables S8, S9 and S10) also give almost the same output.

**Fig. 7:**
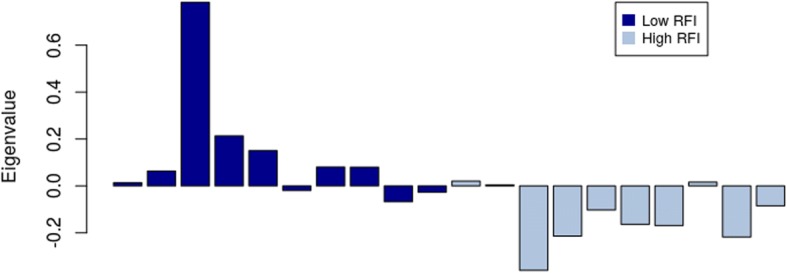
Module eigengene (y-axis) across samples (x-axis) from the lightsteelblue1 module (associated to RFI)

The module eigengene for the lightsteelblue1 module shows has an average expression profile that is lower in high RFI individuals (Fig. 7).

The list of genes with high (MM > 0.8) in the lightsteelblue1 module is presented in Table 2.

**Table 2:** List of the top hub genes generated from (MM > 0.8) in the lightsteelblue1 module in Jersey cows

Ensembl gene ID	Gene name	Module membership	Gene significance
ENSBTAG00000000431	*TRDC*	0.858	−0.411
ENSBTAG00000000432	*TRAC*	0.860	−0.526
ENSBTAG00000000715		0.889	−0.487
ENSBTAG00000001198		0.810	−0.555
ENSBTAG00000002669	*RASSF4*	0.802	−0.722
ENSBTAG00000003037		0.829	−0.485
ENSBTAG00000004894		0.907	−0.497
ENSBTAG00000004917	*KLRK1*	0.826	−0.437
ENSBTAG00000005628		0.818	−0.490
ENSBTAG00000005892	*ZAP70*	0.864	−0.609
ENSBTAG00000006452	*CD3D*	0.900	−0.494
ENSBTAG00000006552	*LAMP3*	0.827	−0.501
ENSBTAG00000007191	*CCL5*	0.909	−0.480
ENSBTAG00000008401	*PFKFB3*	0.808	−0.547
ENSBTAG00000009381	*LCP2*	0.857	−0.654
ENSBTAG00000012695	*LCK*	0.852	−0.510
ENSBTAG00000013730	*CD5*	0.857	−0.403
ENSBTAG00000014725	*CD27*	0.822	−0.474
ENSBTAG00000015708	*CXCR6*	0.879	−0.469
ENSBTAG00000015710	*CD3E*	0.875	−0.537
ENSBTAG00000017256	*CD2*	0.914	−0.474
ENSBTAG00000019403	*MALSU1*	0.800	−0.536
ENSBTAG00000020904	*JAK3*	0.857	−0.439
ENSBTAG00000027246	*UBD*	0.888	−0.621
ENSBTAG00000030426		0.889	−0.379
ENSBTAG00000037510		0.853	−0.433
ENSBTAG00000038639	*CXCL9*	0.906	−0.425
ENSBTAG00000039588		0.815	−0.535
ENSBTAG00000047988		0.842	−0.365

## Discussion

WGCNA identified groups of co-expressed genes that are expected to perform the same biological functions and affect RFI. From the MTR, we tested the modules that were significantly correlated to the focus traits (RFI and diet). However, only the most significant module had any interesting biological meaning associated with the traits (one module in each breed). Hence, only the most biologically meaningful modules were further analysed and discussed.

For Holstein cows, we identified pathways and upstream regulators related to steroid biosynthesis, lipid metabolism, cholesterol metabolism and production in salmon module. In particular, we identified the activation of cholesterol and lipid synthesis in high RFI cows. There was a tendency for these three mechanisms to be activated in the datasets, which is consistent with the idea that high synthesis of fat is correlated with the loss of energy used in milk production in dairy cows, resulting in less feed efficient animals [37]. This finding is also consistent with previous studies that associated high fat deposition with high RFI animals [6, 38]. The magenta module was significantly associated with diet and involved the energy consumption and regulation of glucose.

For Jersey cows, the lightsteelblue1 module was enriched for immune system-related functions. Interestingly, the upstream regulators for the genes in the lightsteelblue1 module (IFNG and IL10RA) were also related to the immune system. In particular, the immune system in high RFI group was activated. Thus, the activation of the immune system leads to low feed efficiency, which is consistent with previous studies [19, 39].

These findings are supported by evidence from the co-expression network analysis of both breeds.

### Co-expressed networks in Holstein cows

The functional enrichment analysis determined that the module identified in Holstein cows was involved in cholesterol biosynthesis, steroid biosynthesis, lipid biosynthesis and fatty acid metabolism.

From the most significant pathways, cholesterol biosynthesis has previously been discussed, as its related genes are important in the RFI. The cholesterol biosynthetic pathway is responsible for the variability of cholesterol levels in cells [40]. This module was also enriched for lipid biosynthesis. Interestingly, the levels of cholesterol and lipids have previously been positively associated with RFI in beef cattle [41].

Many genes in this modules have previously been associated with feed efficiency, [39]. For example, Acetyl-CoA carboxylase alpha (*ACACA*), Acetyl-CoA Acetyltransferase 2 (*ACAT2)*, and fatty acid synthase (*FASN)* genes in the modules are key genes in cholesterol biosynthesis, organic hydroxy compound metabolism, collagen fibril organization, steroid biosynthesis, astral microtubule organization, protein oligomerization and oxidoreductase activity, acting on the CH-CH group of donors and NAD or NADP as an acceptor. *ACACA* and *FASN* were found to be differentially expressed and co-expressed in other feed efficiency-related studies [22, 39, 42]. The main function of *FASN* is to catalyse the synthesis of palmitate from acetyl-CoA and malonyl-CoA, in the presence of NADPH, into long-chain saturated fatty acids. Hence, these genes have a tendency to affect the feed efficiency in Holstein cows. In addition, many studies have discussed the involvement of several genes included in the modules that we identified in the present study (*CYP7A1, ACACA, FASN*) [39, 43]. The presence of *ACAT2* is also interesting because the product of this gene is involved in lipid metabolism [44].

Other feed efficiency studies, for example, in pigs, have previously observed that lipogenesis and steroidogenesis in liver tissue are closely related to feed efficiency [22, 45], confirming previous observations in the differential expression analysis of this dataset [26].

In Holstein cows, we identified ATP7B as a top upstream regulator for the salmon module. This protein uses energy in the molecule adenosine triphosphate (ATP), which is responsible for the transport of metals into and out of cells using the energy stored in the molecule adenosine triphosphate (ATP). ATP7B appears to be activated in high RFI (low FE). Hoogeveen et al. (1995) [46] stated that the deficiency of copper in rats would increase the utilization of fat in rats. Hence, this finding suggests a relationship when ATP7B is activated, which potentially reflects the deposition of fats. Consistent with the present study, the high RFI cow shows the activation of ATP7B. This upstream regulator shows a relationship with regulating the fat consumption. Although it is not straightforward, the presence of the gene reflects the consumption of fat and indirectly affects the fat composition [47].

In the present study, cholesterol synthesis was activated in the IPA upstream regulator analysis. Furthermore, the activation of lipid metabolism in the disease function analysis supports the evidence from the GO term and pathway analyses. As lipid and cholesterol metabolism, and fat synthesis in particular, are activated in the high RFI group, we can assume that the high RFI group is inefficient in converting fat to energy. Hence, animals with high RFI (low FE) have high levels of cholesterol and fat in the body [48]. This finding is also consistent with Arthur et al. [49], who reported the positive relationship between RFI and average back fat in beef carcasses.

Interestingly, when fed a high or low concentrate diet, triglyceride homeostasis was the top GO biological process, which might be the result of the high energy or low energy diet. A previous study reported that controlled diet (with fructose and glucose) significantly affects the triglyceride levels [50].

Generally, based on the results obtained from the functional enrichment analysis for the Holstein breed, the most important GO terms, KEGG pathways and upstream regulators involved were related to steroid biosynthesis, cholesterol biosynthesis, lipid biosynthesis and triglyceride homeostasis. These findings show that the feed efficiency in Holstein cows is strictly associated with the regulation of energy via lipid and cholesterol metabolism.

### Co-expressed networks in Jersey cows

The most significant pathways in Jersey cows were positive regulation of interferon-gamma production, lymphocyte differentiation, side of membrane, natural killer cell-mediated cytotoxicity and primary immunodeficiency. Interestingly, these most summarized pathways were related to the immune system. From the IPA upstream regulator and diseases function analysis, the immune system related functions were activated in the high RFI group.

Several studies also suggested that the involvement of the immune system would affect the feed efficiency [51, 52]. For example, [19, 27] discussed important findings but in different species and breeds. Kristina et al. [39] discovered an increase in the inflammatory response of the progeny of low RFI sires, which is consistent with the results of the present study. The type of diet might also affect the immune response. For example, Ametaj et al. [53] reported that the feeding of high concentrate feeds affects several inflammatory responses in feedlot steers. However, in the present study, no significant effect from the different type of concentrate diet in Jersey cows was observed. This finding might reflect the different populations and different breeds, as dairy cattle convert their nutrients into different products with respect to beef cattle [54]. Although, many other studies relate their findings with the importance of the immune system in RFI and feed efficiency, few studies have been conducted in dairy cattle [19, 20, 39].

Furthermore, these significant GO terms and pathways were also supported by the findings from upstream regulator analysis through IPA®. The top upstream regulator in Jersey cows is Interferon Gamma (IFNG), which has an interesting relationship to interactions among nutrition, metabolism, and the immune system [55]. This gene encodes a soluble cytokine that is a member of the type II interferon class. IFNG was predicted to be inhibited in high RFI Jersey cows. This protein is secreted from cells of both the innate and adaptive immune systems. IFNG is important in the system because it directly inhibits viral replication. The down-regulation of this cytokine in the high RFI group in Jersey cows might affect the feed efficiency. Thus, IFNG plays an important role in regulating immune systems in animals. Another interesting upstream regulator in Jersey cows is IL10RA (Interleukin 10 Receptor Subunit Alpha), which was predicted to be activated. *IL10RA* is a receptor with anti-inflammatory properties [56]. The activation of this gene might result in inhibition of the synthesis of pro-inflammatory cytokines. Reynolds et al. [57] reported that IL10RA was differentially expressed in rumen papillae of divergent average daily gain steers and these authors showed a negative association between the inflammatory response and feed efficiency. Thus, the activation of IL10RA in the high RFI group would reflect the inflammatory response in Jersey cows.

We further speculate that, based on the results obtained in the Jersey breed, the most important GO terms, KEGG pathways and upstream regulators were related to the immune system. Jersey cows have many co-expressed genes that relate to the immune system to regulate feed utilization. It is likely that in Jersey cows, immunity plays a key role in substituting feed nutrient into milk and milk components. The immune response plays an important role in energy balance during milk production in dairy cows.

### Comparison of RFI associated modules between Holstein and Jersey cows

In the datasets analysed in the present study, the most significant module associated with RFI differed between the Holstein and Jersey breeds. Furthermore, these modules were enriched for different sets of biological processes. This evidence suggests that the Holstein cow system is more reactive towards steroid biosynthesis, while Jersey cows have more reactions in their immune systems. Several studies have reported the importance of the lipid and cholesterol metabolism and immune system related functions in feed efficiency traits in farm animals, likely reflecting the complex role of the liver in regulating the nutrient uptake [58].

The hub genes of the modules identified in the present study represent potential candidate genes for RFI. These findings might provide additional information and new insights into the biological processes that are associated with RFI in these two main dairy breeds. Thus, we speculated that in this study population, the liver transcriptomics profiles of the two main dairy breeds are involved in two different biological processes. However, a comparative feed efficiency study reported similar results in terms of digestibility and ratios of milk to body weight and feed intake between Holstein and Jersey cows [12]. The sample size of the present study did not enable confirmation of whether the identified biological processes are breed specific. To confirm this notion, the set of genes should be validated in other cows using qPCR to confirm whether the expression patterns conform to different RFI-diet groups, which is out of the scope of the present study. In addition, a common limitation of static gene co-expression studies is the impossibility to decide if the identified modules are causing variation in the trait analysed or if their co-expression is a consequence of the variation observed for trait. Consequently, in this study we never talk about causality. Further study such as eQTL mapping (data integration between transcriptomics and genomics) could help in understanding better causality between gene expression and trait variation.

## Conclusion

In conclusion, the co-expression network analysis revealed important genes and pathways in the liver that are involved in feed efficiency (RFI). In Holstein cows, the overall results showed that genes and upstream regulators such as ATP7b in RFI-associated modules that were co-expressed were primarily related to steroid and lipid biosynthesis. The results show that high RFI Holstein cows have a high lipid and cholesterol metabolism. The co-expressed genes associated with treatment diet were involved in triglyceride homeostasis. We observed different patterns of co-expressed genes involved in Jersey cows for which most of the co-expressed genes associated with RFI were related to the immune system in the most significant module. The upstream regulators IFNG and ILR10 that were predicted to be inhibited and activated, respectively, were closely associated with the immune system in Jersey cows. A high RFI Jersey cow tends to have a higher response to inflammation. The information of the functional enrichment from the analysis of co-expressed genes provides a better understanding of the mechanisms controlling RFI in Holstein and Jersey cows. Thus, the present study paves the way for the development of biomarkers for feed efficiency in dairy cattle.

## Additional file

Additional file 1: **Table S1 and S2.** Multiple testing corrections for the module trait relationship using Benjamini-Hochberg (BH) method. **Table S3 and S4.** ClueGO analysis output. **Table S5-S10.** STRING 10 analysis for salmon module in Holstein and lightsteelblue1 module in Jersey. **Table S11 and S12.** Upstream regulators from IPA® analysis. **Table S13-S15.** Disease and functions for most significant modules. (DOCX 65 kb)

## Abbreviations

BH: Benjamini-Hochberg
cDNA: Complementary DNA
DM: Dry Matter
FE: Feed Efficiency
GO: Gene Ontology
GS: Gene Significance
HC: High Concentrate
IPA®: Ingenuity® Pathway Analysis
KEGG: Kyoto Encyclopedia of Genes and Genomes
LC: Low Concentrate
MM: Module Membership
mRNA: Messenger RNA
MTR: Module Trait Relationship
RFI: Residual Feed Intake
RNA-seq: RNA-sequencing
TOM: Topological Overlap Measure
WGCNA: Weighted Gene CO-expression Network Analysis
