# Supplementary material for: Gene co-expression networks from RNA sequencing of dairy cattle identifies genes and pathways affecting feed efficiency
Source: BMC Bioinformatics. 2018 Dec 17;19:513. doi: 10.1186/s12859-018-2553-z (PMC6296024; doi:10.1186/s12859-018-2553-z)
Supplement: Supplementary file 1 — Table S1 and S2. Multiple testing corrections for the module trait relationship using Benjamini-Hochberg (BH) method. Table S3 and S4. ClueGO analysis output. Table S5-S10. STRING 10 analysis for salmon module in Holstein and lightsteelblue1 module in Jersey. Table S11 and S12. Upstream regulators from IPA® analysis. Table S13-S15. Disease and functions for most significant modules. (DOCX 65 kb) [file 12859_2018_2553_MOESM1_ESM.docx]

## Additional file

## Gene co-expression networks from RNA sequencing of dairy cattle identifies genes and pathways affecting feed efficiency

Salleh, M.S.^1,2^, Mazzoni, G. ^3,^, Løvendahl, P.^4^, and Kadarmideen, H.N.^5*^

^1^ Department of Veterinary and Animal Sciences, Faculty of Health and Medical Sciences, University of Copenhagen, DK-1870, Frederiksberg C, Denmark

^2^ Department of Animal Science, Faculty of Agriculture, Universiti Putra Malaysia. 43400 UPM Serdang, Selangor, Malaysia

^3^ Department of Bio and Health Informatics, Technical University of Denmark, DK-2800, Kgs. Lyngby, Denmark

^4^ Department of Applied Mathematics and Computer Science, Technical University of Denmark, DK-2800, Kgs. Lyngby, Denmark

^5^ Department of Molecular Biology and Genetics - Center for Quantitative Genetics and Genomics, Aarhus University, AU Foulum, DK-8830, Tjele, Denmark

* Corresponding author

SMS: [surayams@sund.ku.dk](mailto:surayams@sund.ku.dk)

GM: [gianmaz@bioinformatics.dtu.dk](mailto:gianmaz@bioinformatics.dtu.dk)

PL: [peter.lovendahl@mbg.au.dk](mailto:peter.lovendahl@mbg.au.dk)

HNK: [hajak@dtu.dk](mailto:hajak@dtu.dk)

# Multiple testing corrections for the module trait relationship using Benjamini-Hochberg (BH) method

### Table S1 P-value and adjusted P-value for module trait relationship in Hosltein

|  |  | Diet | | RFI | | Parity | |
| --- | --- | --- | --- | --- | --- | --- | --- |
|  | Modules | pvalue | Adj_pval | pvalue | Adj_pval | pvalue | Adj_pval |
| 1 | MEtan | 0.4 | 0.738 | 0.2 | 0.424 | 0.8 | 0.953 |
| 2 | MEskyblue3 | 0.006 | 0.072 | 0.3 | 0.554 | 0.9 | 0.953 |
| 3 | MEyellow4 | 0.4 | 0.738 | 0.7 | 0.900 | 1 | 1.000 |
| 4 | MElightsteelblue | 0.6 | 0.847 | 0.5 | 0.783 | 1 | 1.000 |
| 5 | MEgrey60 | 0.8 | 0.913 | 0.2 | 0.424 | 0.7 | 0.953 |
| 6 | MEthistle2 | 0.5 | 0.837 | 0.1 | 0.300 | 0.8 | 0.953 |
| 7 | MEplum | 0.3 | 0.720 | 0.07 | 0.296 | 0.9 | 0.953 |
| 8 | MElightyellow | 0.9 | 0.913 | 0.005 | 0.130 | 0.8 | 0.953 |
| 9 | MEorange | 0.8 | 0.913 | 0.04 | 0.262 | 0.8 | 0.953 |
| 10 | MEdarkorange | 0.2 | 0.576 | 0.05 | 0.277 | 0.8 | 0.953 |
| 11 | MEorangered4 | 0.8 | 0.913 | 0.009 | 0.130 | 0.9 | 0.953 |
| 12 | MEfloralwhite | 0.08 | 0.343 | 0.03 | 0.262 | 0.9 | 0.953 |
| 13 | MEdarkorange2 | 0.1 | 0.343 | 0.07 | 0.296 | 0.8 | 0.953 |
| 14 | MElightcyan1 | 0.02 | 0.131 | 0.05 | 0.277 | 0.9 | 0.953 |
| 15 | MEdarkgrey | 0.2 | 0.576 | 0.2 | 0.424 | 0.8 | 0.953 |
| 16 | MEsaddlebrown | 0.04 | 0.206 | 0.8 | 0.900 | 0.9 | 0.953 |
| 17 | MEdarkslateblue | 0.4 | 0.738 | 0.9 | 0.900 | 0.8 | 0.953 |
| 18 | MEviolet | 0.01 | 0.090 | 0.7 | 0.900 | 0.8 | 0.953 |
| 19 | MEdarkgreen | 1.00E-04 | 0.004 | 0.7 | 0.900 | 0.9 | 0.953 |
| 20 | MEmagenta | 3.00E-05 | 0.002 | 0.3 | 0.554 | 0.6 | 0.953 |
| 21 | MEdarkturquoise | 0.2 | 0.576 | 0.1 | 0.300 | 0.8 | 0.953 |
| 22 | MEdarkmagenta | 4.00E-04 | 0.010 | 0.1 | 0.300 | 0.8 | 0.953 |
| 23 | MEskyblue | 6.00E-04 | 0.011 | 0.2 | 0.424 | 0.8 | 0.953 |
| 24 | MEdarkolivegreen | 0.9 | 0.913 | 0.009 | 0.130 | 0.9 | 0.953 |
| 25 | MEgreenyellow | 0.7 | 0.900 | 0.006 | 0.130 | 0.8 | 0.953 |
| 26 | MEpalevioletred3 | 0.6 | 0.847 | 0.07 | 0.296 | 0.8 | 0.953 |
| 27 | MEhoneydew1 | 0.4 | 0.738 | 0.02 | 0.206 | 0.9 | 0.953 |
| 28 | MEsalmon | 0.8 | 0.913 | 0.001 | 0.072 | 1 | 1.000 |
| 29 | MEgreen | 0.4 | 0.738 | 0.02 | 0.206 | 0.8 | 0.953 |
| 30 | MElightsteelblue1 | 0.4 | 0.738 | 0.9 | 0.900 | 0.8 | 0.953 |
| 31 | MEbrown | 0.6 | 0.847 | 0.5 | 0.783 | 0.8 | 0.953 |
| 32 | MEbrown4 | 0.6 | 0.847 | 0.2 | 0.424 | 0.8 | 0.953 |
| 33 | MEpurple | 0.7 | 0.900 | 0.2 | 0.424 | 0.8 | 0.953 |
| 34 | MEskyblue2 | 0.6 | 0.847 | 0.2 | 0.424 | 0.8 | 0.953 |
| 35 | MElightcyan | 0.1 | 0.343 | 0.1 | 0.300 | 0.8 | 0.953 |
| 36 | MEthistle1 | 0.1 | 0.343 | 0.09 | 0.300 | 0.7 | 0.953 |
| 37 | MEred | 0.03 | 0.166 | 0.1 | 0.300 | 0.8 | 0.953 |
| 38 | MElavenderblush3 | 0.005 | 0.072 | 0.9 | 0.900 | 0.7 | 0.953 |
| 39 | MEpaleturquoise | 0.02 | 0.131 | 0.2 | 0.424 | 0.8 | 0.953 |
| 40 | MEmediumpurple3 | 0.9 | 0.913 | 0.5 | 0.783 | 0.8 | 0.953 |
| 41 | MEcoral2 | 0.8 | 0.913 | 0.2 | 0.424 | 0.8 | 0.953 |
| 42 | MElightcoral | 0.2 | 0.576 | 0.2 | 0.424 | 0.8 | 0.953 |
| 43 | MEdarkred | 0.7 | 0.900 | 0.3 | 0.554 | 0.8 | 0.953 |
| 44 | MEplum2 | 0.05 | 0.240 | 0.4 | 0.702 | 0.9 | 0.953 |
| 45 | MEmediumpurple2 | 0.5 | 0.837 | 0.8 | 0.900 | 0.8 | 0.953 |
| 46 | MEdarkseagreen4 | 0.4 | 0.738 | 0.9 | 0.900 | 0.8 | 0.953 |
| 47 | MEmidnightblue | 0.6 | 0.847 | 0.4 | 0.702 | 0.8 | 0.953 |
| 48 | MEmaroon | 0.7 | 0.900 | 0.5 | 0.783 | 0.9 | 0.953 |
| 49 | MEyellow | 0.5 | 0.837 | 0.6 | 0.882 | 0.8 | 0.953 |
| 50 | MEcyan | 0.09 | 0.343 | 0.9 | 0.900 | 0.8 | 0.953 |
| 51 | MEturquoise | 0.3 | 0.720 | 0.7 | 0.900 | 0.8 | 0.953 |
| 52 | MEcoral1 | 0.01 | 0.090 | 0.6 | 0.882 | 0.9 | 0.953 |
| 53 | MEplum1 | 0.4 | 0.738 | 0.9 | 0.900 | 0.9 | 0.953 |
| 54 | MEsalmon4 | 1 | 1.000 | 0.04 | 0.262 | 0.8 | 0.953 |
| 55 | MEsienna3 | 0.6 | 0.847 | 0.06 | 0.296 | 0.8 | 0.953 |
| 56 | MEantiquewhite4 | 0.7 | 0.900 | 0.04 | 0.262 | 0.9 | 0.953 |
| 57 | MEivory | 0.9 | 0.913 | 0.3 | 0.554 | 0.9 | 0.953 |
| 58 | MEpink | 0.6 | 0.847 | 0.6 | 0.882 | 0.9 | 0.953 |
| 59 | MEnavajowhite2 | 0.03 | 0.166 | 0.9 | 0.900 | 0.9 | 0.953 |
| 60 | MEorangered3 | 0.9 | 0.913 | 0.9 | 0.900 | 0.9 | 0.953 |
| 61 | MElightpink4 | 0.4 | 0.738 | 0.9 | 0.900 | 0.9 | 0.953 |
| 62 | MEskyblue1 | 0.8 | 0.913 | 0.8 | 0.900 | 0.9 | 0.953 |
| 63 | MEblack | 0.3 | 0.720 | 0.7 | 0.900 | 0.8 | 0.953 |
| 64 | MEblue | 0.9 | 0.913 | 0.9 | 0.900 | 0.8 | 0.953 |
| 65 | MEsteelblue | 0.9 | 0.913 | 0.5 | 0.783 | 0.8 | 0.953 |
| 66 | MElightgreen | 0.1 | 0.343 | 0.8 | 0.900 | 0.7 | 0.953 |
| 67 | MEmediumorchid | 0.02 | 0.131 | 0.9 | 0.900 | 0.7 | 0.953 |
| 68 | MEyellowgreen | 0.5 | 0.837 | 0.1 | 0.300 | 0.8 | 0.953 |
| 69 | MEbisque4 | 0.3 | 0.720 | 0.8 | 0.900 | 0.8 | 0.953 |
| 70 | MEroyalblue | 0.9 | 0.913 | 0.9 | 0.900 | 0.8 | 0.953 |
| 71 | MEwhite | 0.3 | 0.720 | 0.9 | 0.900 | 0.8 | 0.953 |
| 72 | MEgrey | 0.8 | 0.913 | 0.3 | 0.554 | 1 | 1.000 |

### Table S2 P-value and adjusted P-value for module trait relationship in Jersey

|  | Modules | Diet | | RFI | | Parity | |
| --- | --- | --- | --- | --- | --- | --- | --- |
|  |  | pvalue | Adj pval (BH) | pvalue | Adj pval (BH) | pvalue | Adj pval (BH) |
| 1 | MEpalevioletred3 | 0.1 | 0.492 | 0.02 | 0.421 | 1 | 1.000 |
| 2 | MEthistle2 | 0.6 | 0.843 | 0.6 | 0.823 | 0.2 | 0.513 |
| 3 | MEbisque4 | 0.1 | 0.492 | 0.4 | 0.715 | 0.9 | 0.983 |
| 4 | MEmediumpurple3 | 0.1 | 0.492 | 0.4 | 0.715 | 0.9 | 0.983 |
| 5 | MEthistle1 | 0.6 | 0.843 | 0.04 | 0.421 | 0.9 | 0.983 |
| 6 | MEsaddlebrown | 0.1 | 0.492 | 0.5 | 0.756 | 0.06 | 0.253 |
| 7 | MEsalmon4 | 0.1 | 0.492 | 0.1 | 0.421 | 0.06 | 0.253 |
| 8 | MEbrown4 | 0.2 | 0.708 | 0.08 | 0.421 | 0.4 | 0.908 |
| 9 | MEturquoise | 0.1 | 0.492 | 0.2 | 0.492 | 0.9 | 0.983 |
| 10 | MEpaleturquoise | 0.06 | 0.492 | 0.5 | 0.756 | 0.6 | 0.983 |
| 11 | MElightsteelblue1 | 0.8 | 0.908 | 0.009 | 0.421 | 0.3 | 0.708 |
| 12 | MEskyblue3 | 0.3 | 0.708 | 0.2 | 0.492 | 0.8 | 0.983 |
| 13 | MEsteelblue | 0.5 | 0.843 | 0.2 | 0.492 | 0.8 | 0.983 |
| 14 | MEdarkgreen | 0.2 | 0.708 | 0.2 | 0.492 | 0.5 | 0.983 |
| 15 | MEdarkorange2 | 0.9 | 0.948 | 0.4 | 0.715 | 0.6 | 0.983 |
| 16 | MEfloralwhite | 0.5 | 0.843 | 0.07 | 0.421 | 0.09 | 0.295 |
| 17 | MEplum1 | 0.1 | 0.492 | 0.3 | 0.656 | 0.6 | 0.983 |
| 18 | MEdarkslateblue | 0.7 | 0.843 | 0.5 | 0.756 | 0.1 | 0.295 |
| 19 | MEmidnightblue | 0.3 | 0.708 | 0.2 | 0.492 | 0.6 | 0.983 |
| 20 | MElightpink4 | 0.3 | 0.708 | 0.3 | 0.656 | 0.6 | 0.983 |
| 21 | MEbrown | 1 | 1.000 | 0.2 | 0.492 | 0.3 | 0.708 |
| 22 | MElightgreen | 0.3 | 0.708 | 0.9 | 0.900 | 0.5 | 0.983 |
| 23 | MEyellowgreen | 0.6 | 0.843 | 0.7 | 0.858 | 0.5 | 0.983 |
| 24 | MEdarkmagenta | 0.3 | 0.708 | 0.6 | 0.823 | 1 | 1.000 |
| 25 | MEmagenta | 0.5 | 0.843 | 0.2 | 0.492 | 1 | 1.000 |
| 26 | MEdarkolivegreen | 0.3 | 0.708 | 0.8 | 0.858 | 0.9 | 0.983 |
| 27 | MEtan | 0.6 | 0.843 | 0.6 | 0.823 | 0.9 | 0.983 |
| 28 | MEgreenyellow | 0.5 | 0.843 | 0.06 | 0.421 | 0.9 | 0.983 |
| 29 | MEhoneydew1 | 0.7 | 0.843 | 0.06 | 0.421 | 0.8 | 0.983 |
| 30 | MEivory | 0.1 | 0.492 | 0.3 | 0.656 | 0.03 | 0.253 |
| 31 | MElightcyan | 1 | 1.000 | 0.2 | 0.492 | 0.03 | 0.253 |
| 32 | MEorange | 0.7 | 0.843 | 0.1 | 0.421 | 0.04 | 0.253 |
| 33 | MEorangered4 | 0.2 | 0.708 | 0.7 | 0.858 | 0.07 | 0.275 |
| 34 | MElightcyan1 | 0.5 | 0.843 | 0.8 | 0.858 | 0.05 | 0.253 |
| 35 | MEpurple | 0.5 | 0.843 | 0.8 | 0.858 | 0.06 | 0.253 |
| 36 | MEcyan | 0.5 | 0.843 | 0.9 | 0.900 | 1 | 1.000 |
| 37 | MElightyellow | 0.9 | 0.948 | 0.6 | 0.823 | 1 | 1.000 |
| 38 | MEnavajowhite2 | 0.05 | 0.492 | 0.7 | 0.858 | 0.8 | 0.983 |
| 39 | MEdarkgrey | 0.7 | 0.843 | 0.2 | 0.492 | 0.9 | 0.983 |
| 40 | MEskyblue | 0.7 | 0.843 | 0.4 | 0.715 | 0.9 | 0.983 |
| 41 | MEroyalblue | 0.3 | 0.708 | 0.8 | 0.858 | 0.03 | 0.253 |
| 42 | MEwhite | 0.9 | 0.948 | 0.8 | 0.858 | 0.05 | 0.253 |
| 43 | MEblack | 0.9 | 0.948 | 0.9 | 0.900 | 0.03 | 0.253 |
| 44 | MElavenderblush3 | 0.3 | 0.708 | 0.7 | 0.858 | 0.04 | 0.253 |
| 45 | MEsalmon | 0.7 | 0.843 | 0.9 | 0.900 | 0.9 | 0.983 |
| 46 | MEdarkturquoise | 0.5 | 0.843 | 0.4 | 0.715 | 0.9 | 0.983 |
| 47 | MEmaroon | 0.3 | 0.708 | 0.5 | 0.756 | 0.9 | 0.983 |
| 48 | MEred | 0.7 | 0.843 | 0.2 | 0.492 | 0.5 | 0.983 |
| 49 | MEblue | 0.6 | 0.843 | 0.5 | 0.756 | 0.1 | 0.295 |
| 50 | MEpink | 0.8 | 0.908 | 0.8 | 0.858 | 0.2 | 0.513 |
| 51 | MEyellow | 0.6 | 0.843 | 0.8 | 0.858 | 0.8 | 0.983 |
| 52 | MEgreen | 1 | 1.000 | 0.09 | 0.421 | 0.2 | 0.513 |
| 53 | MEplum2 | 0.04 | 0.492 | 0.1 | 0.421 | 0.7 | 0.983 |
| 54 | MEdarkorange | 0.5 | 0.843 | 0.7 | 0.858 | 0.1 | 0.295 |
| 55 | MEdarkred | 0.6 | 0.843 | 0.4 | 0.715 | 0.1 | 0.295 |
| 56 | MEsienna3 | 0.2 | 0.708 | 0.03 | 0.421 | 0.05 | 0.253 |
| 57 | MEgrey60 | 0.8 | 0.908 | 0.1 | 0.421 | 0.04 | 0.253 |
| 58 | MEviolet | 0.04 | 0.492 | 0.5 | 0.756 | 0.04 | 0.253 |
| 59 | MEgrey | 0.5 | 0.843 | 0.1 | 0.421 | 0.6 | 0.983 |

# ClueGO analysis output

### Table S3 Significant (p<0.05) GO term and pathways in salmon module in Holstein

| GO term function | PValue Corrected with Benjamini-Hochberg | Number of genes |
| --- | --- | --- |
| cholesterol biosynthetic process | 3.1E-04 | 27 |
| GTPase binding | 3.8E-04 | 12 |
| organic hydroxy compound metabolic process | 4.4E-04 | 16 |
| collagen fibril organization | 5.8E-04 | 10 |
| microtubule organizing center | 6.4E-04 | 22 |
| Steroid biosynthesis | 7.6E-04 | 34 |
| cytoskeletal part | 8.0E-04 | 30 |
| astral microtubule organization | 9.7E-04 | 50 |
| Leukocyte transendothelial migration | 1.4E-03 | 7 |
| small GTPase mediated signal transduction | 5.0E-03 | 14 |
| oxidoreductase activity, acting on the CH-CH group of donors, NAD or NADP as acceptor | 5.2E-03 | 3 |
| Measles | 1.1E-02 | 7 |
| oxidoreductase activity, acting on paired donors, with incorporation or reduction of molecular oxygen, NAD(P)H as one donor, and incorporation of one atom of oxygen | 1.2E-02 | 5 |
| sarcomere | 1.4E-02 | 4 |
| Protein oligomerization | 1.9E-02 | 8 |
| Glycerolipid metabolism | 2.2E-02 | 3 |
| lung alveolus development | 2.2E-02 | 3 |
| phagocytic vesicle | 2.6E-02 | 3 |
| cellular response to nitrogen compound | 3.0E-02 | 8 |
| solute:cation symporter activity | 3.1E-02 | 3 |

### Table S4 Significant (p<0.05) GO term and pathways in lightsteelblue1 module in Jersey

| GO term function | PValue Corrected with Benjamini-Hochberg | Number of genes |
| --- | --- | --- |
| positive regulation of interferon-gamma production | 5.50E-10 | 11 |
| lymphocyte differentiation | 6,43E-09 | 13 |
| side of membrane | 2.47E-08 | 27 |
| Natural killer cell mediated cytotoxicity | 2,89E-08 | 15 |
| external side of plasma membrane | 6.97E-06 | 11 |
| second-messenger-mediated signaling | 6.10E-05 | 5 |
| Cell adhesion molecules (CAMs) | 4.36E-03 | 3 |

# STRING 10 analysis for salmon module in Holstein and lightsteelblue1 module in Jersey

## Holstein

### Table S5 Biological Process

| #pathway ID | pathway description | observed gene count | FDR | matching proteins in your network (labels) |
| --- | --- | --- | --- | --- |
| GO.0006695 | cholesterol biosynthetic process | 8 | 1.53E-09 | *CYP51A1,DHCR7,FDFT1,HMGCR,IDI1,MVD,MVK,NSDHL* |
| GO.0006694 | steroid biosynthetic process | 9 | 8.49E-09 | *CYP51A1,DHCR7,FDFT1,HMGCR,IDI1,LSS,MVD,MVK,NSDHL* |
| GO.0008610 | lipid biosynthetic process | 12 | 1.84E-06 | *ACACA,ACLY,CYP51A1,DHCR7,FASN,FDFT1,GPAM,HMGCR,IDI1,LSS,MVK,NSDHL* |
| GO.0044283 | small molecule biosynthetic process | 10 | 0.000173 | *ACACA,CYP51A1,DHCR7,FASN,FDFT1,HMGCR,IDI1,MVD,MVK,NSDHL* |
| GO.0006629 | lipid metabolic process | 13 | 0.00156 | *ACACA,ACLY,CYP51A1,DHCR7,FASN,FDFT1,GPAM,HMGCR,IDI1,LSS,MVK,NCF1,NSDHL* |
| GO.0008299 | isoprenoid biosynthetic process | 4 | 0.00368 | *FDFT1,HMGCR,IDI1,MVK* |
| GO.0022614 | membrane to membrane docking | 2 | 0.0468 | *EZR,MSN* |

### Table S6 Cellular Component

| #pathway ID | pathway description | observed gene count | FDR | matching proteins in your network (labels) |
| --- | --- | --- | --- | --- |
| GO.0001931 | uropod | 2 | 0.0296 | *EZR,MSN* |
| GO.0031254 | cell trailing edge | 2 | 0.0296 | *EZR,MSN* |
| GO.0036064 | ciliary basal body | 4 | 0.0296 | *CETN2,EZR,POC1A,PSEN2* |
| GO.0016324 | apical plasma membrane | 5 | 0.0365 | *AMOTL2,CLDN1,EZR,MSN,PSEN2* |
| GO.0044444 | cytoplasmic part | 35 | 0.0418 | *ACACA,ACLY,ACTR3,AMOTL2,BAIAP2,CETN2,CKB,COPG2,CTSK,CYP51A1,DHCR7,EIF2C2,FAM109B,FASN,FDFT1,GPAM,HMGCR,IDI1,IKBIP,LSS,MAP1S,MIA3,MOSPD1,MVD,MVK,NCF1,NSDHL,POC1A,RAB11A,RAB8B,SHOC2,SLC1A4,SLC25A37,SLC35A3,WDR44* |

### Table S7 KEGG pathways

| #pathway ID | pathway description | observed gene count | FDR | matching proteins in your network (labels) |
| --- | --- | --- | --- | --- |
| 100 | Steroid biosynthesis | 9 | 2.85E-12 | *CYP51A1,DHCR24,DHCR7,FDFT1,HSD17B7,LSS,MSMO1,NSDHL,SQLE* |
| 900 | Terpenoid backbone biosynthesis | 6 | 1.35E-06 | *ACAT2,HMGCR,HMGCS1,IDI1,MVD,MVK* |
| 1100 | Metabolic pathways | 29 | 2.29E-05 | *ACACA,ACAT2,ACLY,ACSS2,CKB,CSAD,CYP51A1,CYP7A1,DGKD,DHCR24,DHCR7,ELOVL6,FASN,FDFT1,GPAM,HMGCR,HMGCS1,HSD17B7,IDI1,IDUA,LSS,MBOAT2,MSMO1,MVD,MVK,NSDHL,PHOSPHO2,RDH11,SQLE* |
| 1212 | Fatty acid metabolism | 5 | 0.00386 | *ACACA,ACAT2,ELOVL6,FADS1,FASN* |
| 4670 | Leukocyte transendothelial migration | 7 | 0.00386 | *CLDN1,EZR,GNAI1,MSN,NCF1,PIK3CG,RAPGEF4* |

## Jersey

### Table S8 Biological Process

| #pathway ID | pathway description | observed gene count | FDR | matching proteins in your network (labels) |
| --- | --- | --- | --- | --- |
| GO.0050870 | positive regulation of T cell activation | 4 | 1.03E-02 | *CCL5,CD3E,CD5,SASH3* |
| GO.0002684 | positive regulation of immune system process | 6 | 1.16E-02 | *Bt.87330,CCL5,CD3E,CD5,CXCL9,SASH3* |
| GO.0070098 | chemokine-mediated signaling pathway | 3 | 1.62E-02 | *CCL5,CCR5,CXCL9* |
| GO.0002376 | immune system process | 7 | 0.0203 | *CCL5,CCR5,CD3D,CD3E,CD5,PSMB8,PSMB9* |
| GO.0032753 | positive regulation of interleukin-4 production | 2 | 0.0203 | *CD3E,SASH3* |
| GO.0042102 | positive regulation of T cell proliferation | 3 | 0.0203 | *CCL5,CD3E,SASH3* |
| GO.0045061 | thymic T cell selection | 2 | 0.0203 | *CD3D,CD3E* |
| GO.0016337 | single organismal cell-cell adhesion | 4 | 0.024 | *CCL5,CD3D,CD3E,ICAM3* |
| GO.0031295 | T cell costimulation | 2 | 0.0269 | *CD3E,CD5* |
| GO.0007155 | cell adhesion | 5 | 0.0476 | *CCL5,CD3D,CD3E,CD96,ICAM3* |

### Table S9 Cellular Component

| #pathway ID | pathway description | observed gene count | FDR | matching proteins in your network (labels) |
| --- | --- | --- | --- | --- |
| GO.0009897 | external side of plasma membrane | 4 | 3.51E-03 | *CCR5,CD3E,CD5,CXCL9* |
| GO.0042105 | alpha-beta T cell receptor complex | 2 | 8.53E-03 | *CD3D,CD3E* |
| GO.1990111 | spermatoproteasome complex | 2 | 1.02E-02 | *PSMB8,PSMB9* |

### Table S10 KEGG Pathway

| #pathway ID | pathway description | observed gene count | FDR | matching proteins in your network (labels) |
| --- | --- | --- | --- | --- |
| 4650 | Natural killer cell mediated cytotoxicity | 7 | 1.97E-06 | *CD244,KLRK1,LAT,LCK,LCP2,PRF1,ZAP70* |
| 4660 | T cell receptor signaling pathway | 7 | 1.97E-06 | *CARD11,CD3D,CD3E,LAT,LCK,LCP2,ZAP70* |
| 5340 | Primary immunodeficiency | 4 | 2.21E-04 | *CD3D,CD3E,LCK,ZAP70* |
| 4064 | NF-kappa B signaling pathway | 5 | 0.000279 | *CARD11,LAT,LCK,PTGS2,ZAP70* |
| 4640 | Hematopoietic cell lineage | 4 | 0.00321 | *CD2,CD3D,CD3E,CD5* |
| 5166 | HTLV-I infection | 6 | 0.00321 | *CD3D,CD3E,HLA-DOA,JAK3,JSP.1,LCK* |
| 4062 | Chemokine signaling pathway | 5 | 0.00428 | *CCL5,CCR5,CXCL9,CXCR6,JAK3* |
| 5332 | Graft-versus-host disease | 3 | 0.00443 | *HLA-DOA,JSP.1,PRF1* |
| 5330 | Allograft rejection | 3 | 0.00583 | *HLA-DOA,JSP.1,PRF1* |
| 4060 | Cytokine-cytokine receptor interaction | 5 | 0.00699 | *CCL5,CCR5,CD27,CXCL9,CXCR6* |
| 4940 | Type I diabetes mellitus | 3 | 0.00699 | *HLA-DOA,JSP.1,PRF1* |
| 5320 | Autoimmune thyroid disease | 3 | 0.00699 | *HLA-DOA,JSP.1,PRF1* |
| 4514 | Cell adhesion molecules (CAMs) | 4 | 0.0122 | *CD2,HLA-DOA,ICAM3,JSP.1* |
| 5416 | Viral myocarditis | 3 | 0.0122 | *HLA-DOA,JSP.1,PRF1* |
| 5203 | Viral carcinogenesis | 4 | 0.0394 | *Bt.87330,CCR5,JAK3,JSP.1* |

# Upstream regulators from IPA® analysis

### Table 11 Upstream regulators for Holstein

| Upstream Regulator | Expr Log Ratio | Molecule Type | Predicted Activation State | Bias-corrected z-score | p-value of overlap | Target molecules in dataset | Mechanistic Network |
| --- | --- | --- | --- | --- | --- | --- | --- |
| ATP7B | 0,162 | transporter | Activated | 2,747 | 1,19E-20 | ACLY,CYP51A1,CYP7A1,ELOVL6,FASN,FDFT1,FDPS,HMGCR,HMGCS1,IDI1,LSS,MSMO1,SQLE,SREBF2 |  |
| POR | 0,113 | enzyme | Inhibited | -2,653 | 2,66E-19 | ACAT2,ACLY,CSAD,CYB5B,CYP51A1,CYP7A1,DHCR24,DHCR7,ELOVL6,FDFT1,FDPS,HMGCR,HMGCS1,IDI1,LSS,MSMO1,MVD,MVK,NSDHL,SQLE,SREBF2 |  |
| cholesterol |  | chemical - endogenous mammalian | Inhibited | -3,898 | 6,09E-18 | ACLY,ACSS2,CTSK,CYB5B,CYP7A1,DHCR7,FADS1,FASN,FCGR2B,FDFT1,FDPS,GNAI1,HMGCR,HMGCS1,IDI1,LYZ,MSMO1,NSDHL,RDH11,SQLE,SREBF2 | 53 (12) |

### Table 12 Upstream regulators for Jersey

| Upstream Regulator | Expr Log Ratio | Molecule Type | Predicted Activation State | Bias-corrected z-score | p-value of overlap | Target molecules in dataset | Mechanistic Network |
| --- | --- | --- | --- | --- | --- | --- | --- |
| IFNG |  | cytokine | Inhibited | -2,068 | 1,43E-06 | CCL5,CCR5,CD2,CXCL9,GBP5,HLA-B,HLA-DOA,JAK3,LAMP3,LCP2,PSMB8,PSMB9,PTGS2,UBD | 20 (12) |
| IL10RA | -0,326 | transmembrane receptor | Activated | 2,526 | 1,04E-04 | CCL5,GBP5,Klrk1,PSMB8,PSMB9,TRPM2 | 12 (4) |
| NKX2-3 |  | transcription regulator | Activated | 2,035 | 1,96E-03 | HLA-B,PSMB8,PSMB9,PTGS2 |  |
| dexamethasone |  | chemical drug | Activated | 2,67 | 5,26E-02 | CCL5,CD3D,CD3E,ICAM3,JAK3,PFKFB3,PTGS2,UBD |  |

# Disease and functions for most significant modules

### Table 13 Diseases and functions in Salmon module in Holstein cows

| Categories | Diseases or Functions Annotation | p-Value | Predicted Activation State | Activation z-score | Molecules | # Molecules |
| --- | --- | --- | --- | --- | --- | --- |
| Lipid Metabolism, Small Molecule Biochemistry, Vitamin and Mineral Metabolism | synthesis of cholesterol | 3,66E-17 | Increased | 2.321 | ACLY,CYP51A1,CYP7A1,DHCR24,DHCR7,FDFT1,FDPS,HMGCR,HSD17B7,HTT,IDI1,LSS,MVK,SREBF2 | 14 |
| Lipid Metabolism, Small Molecule Biochemistry, Vitamin and Mineral Metabolism | synthesis of sterol | 6,08E-17 | Increased | 2.573 | ACAT2,ACLY,CYP51A1,CYP7A1,DHCR24,DHCR7,FDFT1,FDPS,HMGCR,HSD17B7,HTT,IDI1,LSS,MVK,SREBF2 | 15 |
| Lipid Metabolism, Small Molecule Biochemistry, Vitamin and Mineral Metabolism | metabolism of cholesterol | 2,67E-16 | Increased | 2.405 | ACLY,CYP51A1,CYP7A1,DHCR24,DHCR7,FDFT1,FDPS,HMGCR,HSD17B7,HTT,IDI1,LSS,MVK,NSDHL,SQLE,SREBF2 | 16 |
| Lipid Metabolism, Small Molecule Biochemistry | metabolism of membrane lipid derivative | 1,21E-11 | Increased | 2.922 | ACLY,CYP51A1,CYP7A1,DHCR24,DHCR7,FADS1,FASN,FCGR2B,FDFT1,FDPS,GPAM,HMGCR,HSD17B7,HTT,IDI1,LSS,MVK,NSDHL,PIK3CG,RHOQ,SQLE,SREBF2 | 22 |
| Lipid Metabolism, Small Molecule Biochemistry | synthesis of lipid | 3,92E-10 | Increased | 3.616 | ACACA,ACAT2,ACLY,ACSS2,BMP4,CYP51A1,CYP7A1,DGKD,DHCR24,DHCR7,ELOVL6,FADS1,FASN,FCGR2B,FDFT1,FDPS,GPAM,HMGCR,HSD17B7,HTT,IDI1,LSS,MVD,MVK,NCF1,PIK3CG,RHOQ,SREBF2 | 28 |
| Lipid Metabolism, Nucleic Acid Metabolism, Small Molecule Biochemistry | conversion of acyl-coenzyme A | 1,47E-08 | Increased | 2.200 | ACACA,ACLY,ACSS2,FASN,HMGCR | 5 |
| Lipid Metabolism, Small Molecule Biochemistry | conversion of lipid | 5,51E-07 | Increased | 2.967 | ACACA,ACLY,ACSS2,CYB5B,DHCR24,FADS1,FASN,FCGR2B,HMGCR,HTT | 10 |
| Lipid Metabolism, Small Molecule Biochemistry | conversion of fatty acid | 1,30E-06 | Increased | 2.414 | ACACA,ACLY,ACSS2,FADS1,FASN,HMGCR | 6 |
| Cellular Assembly and Organization, Cellular Function and Maintenance | organization of cytoplasm | 1,07E-05 | Increased | 4.531 | ACACA,ACTR3,ANKRD27,BAIAP2,BICDL1,BMP4,CEP120,CETN2,CGN,CHD3,DNAJC13,EVL,EZR,FASN,HTT,IDUA,KIF3B,LCK,LCP1,MAP1S,MAPKAPK5,MSN,PRKCH,PSEN2,PTPRE,RAB11A,RALBP1,RAPGEF4,RHOQ,RYR1,SPTAN1,STK26,WIPF1,YME1L1 | 34 |
| Cellular Assembly and Organization, Cellular Function and Maintenance | organization of cytoskeleton | 2,47E-05 | Increased | 4.507 | ACACA,ACTR3,ANKRD27,BAIAP2,BICDL1,BMP4,CEP120,CETN2,CGN,CHD3,EVL,EZR,FASN,HTT,KIF3B,LCK,LCP1,MAP1S,MAPKAPK5,MSN,PRKCH,PSEN2,PTPRE,RAB11A,RALBP1,RAPGEF4,RHOQ,RYR1,SPTAN1,STK26,WIPF1 | 31 |
| Infectious Diseases | Viral Infection | 4,76E-05 | Increased | 3.270 | ACTR3,AGO2,CDC40,CYB5B,CYP51A1,FASN,FCGR2B,FDFT1,FDPS,HLA-DOA,HMGCR,HMGCS1,IFIH1,IGHMBP2,KIAA0922,LCK,MAP1S,MYOF,NCF1,PIK3CG,PRKCH,PSEN2,RAB11A,RAB8B,SLU7,SPRY2,SPTAN1,SREBF2,TMC8,TRIM5,TUBB2A,WIPF1,XK,ZNF791 | 34 |
| Cell Morphology, Cellular Assembly and Organization, Cellular Function and Maintenance | formation of cellular protrusions | 6,81E-05 | Increased | 4.031 | ACACA,ACTR3,ANKRD27,BAIAP2,BICDL1,CEP120,CETN2,EZR,FASN,HTT,KIF3B,LCP1,MAP1S,MSN,PSEN2,PTPRE,RAB11A,RAPGEF4,RHOQ,RYR1,STK26,WIPF1 | 22 |
| Cellular Assembly and Organization, Cellular Function and Maintenance | microtubule dynamics | 1,23E-04 | Increased | 4.408 | ACACA,ACTR3,ANKRD27,BAIAP2,BICDL1,BMP4,CEP120,CETN2,CGN,EVL,EZR,FASN,HTT,KIF3B,LCP1,MAP1S,MSN,PRKCH,PSEN2,PTPRE,RAB11A,RAPGEF4,RHOQ,RYR1,STK26,WIPF1 | 26 |
| Infectious Diseases | infection by lentivirus | 1,25E-04 | Increased | 2.458 | ACTR3,CDC40,CYP51A1,FCGR2B,FDFT1,FDPS,HLA-DOA,HMGCR,HMGCS1,IGHMBP2,KIAA0922,MYOF,PRKCH,PSEN2,SLU7,SPTAN1,TRIM5,TUBB2A,ZNF791 | 19 |
| Lipid Metabolism, Molecular Transport, Small Molecule Biochemistry | concentration of sterol | 1,40E-04 | Increased | 2.357 | ACAT2,CYP7A1,DHCR24,DHCR7,FDFT1,GPAM,HMGCR,HTT,KLB,PSEN2,SREBF2 | 11 |
| Organismal Survival | organismal death | 1,46E-04 | Decreased | -4.455 | ACACA,ACLY,AGO2,BMP4,CETN2,CHEK2,CLDN1,COL4A1,CSAD,CYP51A1,CYP7A1,DGKD,DHCR7,E2F3,ELK3,FASN,FCGR2B,FDFT1,GNAI1,HMGCR,HS6ST1,HSD17B7,HTT,IDUA,IFIH1,KIF3B,LCP1,LPAR6,LYZ,MAPKAPK5,MIA3,MSN,NCF1,PIK3CG,PRKCH,PSEN2,RAB11A,RAB8B,RYR1,SHOC2,SPRY2,SREBF2,TCEA1,WIPF1 | 44 |
| Lipid Metabolism, Molecular Transport, Small Molecule Biochemistry | concentration of lipid | 2,07E-04 | Increased | 3.143 | ACACA,ACAT2,ACLY,CYP7A1,DHCR24,DHCR7,E2F3,FASN,FCGR2B,FDFT1,GNAI1,GPAM,HMGCR,HS6ST1,HTT,IGHMBP2,KLB,PIK3CG,PSEN2,SREBF2 | 20 |
| Cell Morphology, Cellular Assembly and Organization, Cellular Function and Maintenance | reorganization of cytoskeleton | 2,59E-04 | Increased | 2.213 | BAIAP2,EZR,LCK,LCP1,MAPKAPK5,MSN,RHOQ,SPTAN1 | 8 |
| Infectious Diseases | HIV infection | 3,53E-04 | Increased | 2.776 | ACTR3,CDC40,CYP51A1,FCGR2B,FDFT1,FDPS,HLA-DOA,HMGCR,HMGCS1,IGHMBP2,KIAA0922,MYOF,PRKCH,PSEN2,SLU7,SPTAN1,TUBB2A,ZNF791 | 18 |
| Lipid Metabolism, Molecular Transport, Small Molecule Biochemistry | concentration of cholesterol | 3,88E-04 | Increased | 2.263 | CYP7A1,DHCR24,DHCR7,FDFT1,GPAM,HMGCR,HTT,KLB,PSEN2,SREBF2 | 10 |
| Cellular Growth and Proliferation, Tissue Development | generation of cells | 1,02E-03 | Increased | 2.328 | ACTR3,AGO2,ANKRD27,BAIAP2,BICDL1,BMP4,CD3D,CGN,COL4A1,DDIAS,DHCR24,EZR,FCGR2B,Fmnl1,HLA-DOA,HS6ST1,HTT,KIF3B,LCK,LOXL2,MAP1S,MIA3,MSN,MYOF,PIK3CG,PRKCH,PSEN2,PTPRE,RAB11A,RALBP1,RAPGEF4,RHOQ,RYR1,SFMBT1,TCF7,WIPF1 | 36 |
| Developmental Disorder | hypoplasia of organ | 1,21E-03 | Decreased | -3.399 | BMP4,CYP51A1,CYTIP,E2F3,HS6ST1,HSD17B7,LCK,PIK3CG,PRKCH,SPRY2,TCEA1,WIPF1 | 12 |
| Lipid Metabolism, Molecular Transport, Small Molecule Biochemistry | quantity of steroid | 1,92E-03 | Increased | 3.157 | ACAT2,CYP7A1,DHCR24,DHCR7,FDFT1,GPAM,HMGCR,HTT,IGHMBP2,KLB,PSEN2,SREBF2 | 12 |
| Lipid Metabolism, Small Molecule Biochemistry | fatty acid metabolism | 2,05E-03 | Increased | 2.423 | ACACA,ACLY,ACSS2,CYP7A1,ELOVL6,FADS1,FASN,FCGR2B,GPAM,HTT,LSS,MSMO1,MSN,NCF1 | 14 |
| Cellular Growth and Proliferation | proliferation of cells | 2,23E-03 | Increased | 2.733 | ACACA,ACLY,AGO2,BAIAP2,BMP4,CEP120,CHEK2,CLDN1,COL4A1,CSNK1G3,CYTIP,DGKD,DHCR24,DHCR7,E2F3,EZR,FADS1,FASN,FCGR2B,FDFT1,GNAI1,GPAM,HMGCR,HS6ST1,HTT,KLB,LCK,LCP1,LOXL2,LRRFIP1,LYZ,MMP11,MVD,MYOF,NCF1,PIK3CG,PIK3IP1,PPP1CB,PRKCH,PSEN2,PTPRE,RAB11A,RAB8B,RALBP1,RHOQ,SKAP2,SPRY2,SPTAN1,STK26,TCF7,THAP12,TMC8,TUBB2A,USP36,WIPF1,YME1L1 | 56 |
| Infectious Diseases | infection of cells | 2,43E-03 | Increased | 2.509 | ACTR3,CDC40,CYB5B,FCGR2B,HLA-DOA,HMGCR,HMGCS1,IGHMBP2,KIAA0922,MYOF,PRKCH,PSEN2,RAB8B,SLU7,SPTAN1,TRIM5,ZNF791 | 17 |
| Developmental Disorder | dysgenesis | 2,62E-03 | Decreased | -3.393 | BMP4,CYP51A1,CYTIP,E2F3,HS6ST1,HSD17B7,LCK,PIK3CG,PRKCH,SLC1A4,SPRY2,TCEA1,WIPF1 | 13 |
| Lipid Metabolism, Small Molecule Biochemistry | metabolism of phospholipid | 4,89E-03 | Increased | 2.142 | CYP7A1,FADS1,FASN,FCGR2B,GPAM,PIK3CG,RHOQ | 7 |
| Cell Death and Survival | cell viability | 4,95E-03 | Increased | 3.511 | ACACA,ACLY,AGO2,BMP4,CDC40,CHEK2,E2F3,EZR,FASN,HTT,IFIH1,LCK,LYZ,PIK3CG,PIK3IP1,PPP1CB,PRKCH,PSEN2,PTPRE,RAB11A,RAPGEF4,SREBF2,TCF7,USP36 | 24 |

### Table 14 Diseases and functions in Magenta module in Holstein cows

| Categories | Diseases or Functions Annotation | p-Value | Predicted Activation State | Activation z-score | Molecules | # Molecules |
| --- | --- | --- | --- | --- | --- | --- |
| Cell Cycle | senescence of cells | 2,14E-04 | Decreased | -2.078 | BHLHE40,BRCA1,DUSP1,FANCD2,GADD45A,MAPK9,NAMPT,NFE2L2,NRAS,PAX8,SRF | 11 |
| Lipid Metabolism, Small Molecule Biochemistry | synthesis of fatty acid | 3,52E-04 | Decreased | -2.066 | ABAT,ACADL,ACSL1,APOA4,APOA5,BRCA1,CD14,ELOVL2,FOXA1,HNF1A,MAPK9,MID1IP1 | 12 |
| Carbohydrate Metabolism | quantity of monosaccharide | 8,66E-04 | Increased | 2.359 | ACSL1,ADIPOR2,ADM,ALDH1A1,APOA4,C1QTNF12,DGAT2,FOXA1,HNF1A,MADD,NRAS,RGS16 | 12 |
| Carbohydrate Metabolism, Molecular Transport, Small Molecule Biochemistry | concentration of D-glucose | 2,41E-03 | Increased | 2.359 | ACSL1,ADIPOR2,ADM,ALDH1A1,APOA4,C1QTNF12,DGAT2,FOXA1,HNF1A,MADD,RGS16 | 11 |
| Carbohydrate Metabolism | uptake of carbohydrate | 2,82E-03 | Decreased | -2.343 | ACSL1,ADM,APOA1,C1QTNF12,CD14,CRAT,HNF1A,MAPK9,NR1I2,SRF | 10 |
| Molecular Transport | export of molecule | 6,68E-03 | Decreased | -2.124 | ACSL1,APOA1,APOA4,APOA5,CD14,CRAT,NR1I2,SLC22A7,SOAT2 | 9 |

### Table 15 Diseases and functions in Lightsteelblue1 module in Jersey cows

| Categories | Diseases or Functions Annotation | p-Value | Predicted Activation State | Activation z-score | Molecules | # Molecules |
| --- | --- | --- | --- | --- | --- | --- |
| Cell-To-Cell Signaling and Interaction, Hematological System Development and Function, Immune Cell Trafficking, Inflammatory Response | activation of leukocytes | 4.66E-21 | Decreased | -2.61 | CARD11,CCL5,CCR5,CD2,CD27,CD3D,CD3E,CD5,CXCL9,GIMAP1-GIMAP5,HLA-DOA,ICAM3,JAK3,Klre1,Klrk1,LAT,LCK,LCP2,PRF1,PSMB8,PSMB9,PTGS2,SASH3,TRPM2,ZAP70 | 25 |
| Hematological System Development and Function, Lymphoid Tissue Structure and Development, Tissue Morphology | quantity of T lymphocytes | 2.03E-14 | Decreased | -3.43 | CARD11,CCL5,CCR5,CD27,CD3D,CD3E,CD5,GIMAP1-GIMAP5,JAK3,Klrk1,LAT,LCK,LCP2,PRF1,PSMB8,PSMB9,SASH3,ZAP70 | 18 |
| Cellular Development, Hematological System Development and Function, Hematopoiesis, Lymphoid Tissue Structure and Development | differentiation of leukocytes | 5.18E-14 | Decreased | -2.07 | CARD11,CCL5,CD2,CD27,CD3D,CD3E,GIMAP1,GIMAP1-GIMAP5,HLA-DOA,JAK3,Klrk1,LAT,LCK,LCP2,PTGS2,SASH3,TRPM2,UBD,ZAP70 | 19 |
| Hematological System Development and Function, Tissue Morphology | quantity of leukocytes | 3.06E-12 | Decreased | -3.44 | CARD11,CCL5,CCR5,CD27,CD3D,CD3E,CD5,CXCR6,GIMAP1-GIMAP5,JAK3,Klrk1,LAT,LCK,LCP2,PRF1,PSMB8,PSMB9,PTGS2,SASH3,ZAP70 | 20 |
| Cell Death and Survival, Cellular Compromise | cytotoxicity of lymphocytes | 9.59E-12 | Decreased | -2.22 | CARD11,CCL5,CD2,CD27,CD5,CD96,Klrk1,LAT,LCK,PRF1 | 10 |
| Cellular Movement, Hematological System Development and Function, Immune Cell Trafficking | Lymphocyte migration | 3.41E-10 | Decreased | -2.04 | CCL5,CCR5,CD2,CD3E,CXCL9,JAK3,LAT,LCK,LCP2,PRF1,PTGS2,ZAP70 | 12 |
| Developmental Disorder, Organismal Injury and Abnormalities | hypoplasia of lymphatic system | 5.04E-10 | Increased | 2.92 | CD3E,GIMAP1-GIMAP5,JAK3,Klrk1,LAT,LCK,LCP2,PRF1,SASH3 | 9 |
| Cell-To-Cell Signaling and Interaction, Hematological System Development and Function | interaction of T lymphocytes | 3.64E-09 | Decreased | -2.20 | CCL5,CCR5,CD2,CXCL9,ICAM3,LCK,LCP2 | 7 |
| Cell Signaling, Molecular Transport, Vitamin and Mineral Metabolism | mobilization of Ca2+ | 4.41E-09 | Decreased | -2.33 | CCL5,CCR5,CD2,CD3E,CD5,CXCL9,CXCR6,LAT,LCK,LCP2,ZAP70 | 11 |
| Developmental Disorder, Immunological Disease, Organismal Injury and Abnormalities | hypoplasia of lymphoid organ | 9.40E-09 | Increased | 2.75 | CD3E,GIMAP1-GIMAP5,JAK3,Klrk1,LAT,LCK,LCP2,PRF1 | 8 |
| Cell-To-Cell Signaling and Interaction, Hematological System Development and Function, Immune Cell Trafficking, Inflammatory Response | activation of natural killer cells | 4.84E-08 | Decreased | -2.19 | CARD11,CCL5,CD2,CD27,Klre1,Klrk1,PRF1 | 7 |
| Cell-To-Cell Signaling and Interaction, Hematological System Development and Function, Immune Cell Trafficking | adhesion of immune cells | 7.06E-08 | Decreased | -2.96 | CCL5,CCR5,CD2,CXCL9,ICAM3,JAK3,LCK,LCP2,PTGS2,ZAP70 | 10 |
| Cellular Development | differentiation of cells | 7.89E-08 | Decreased | -2.27 | ARHGEF3,CARD11,CCL5,CCR5,CD2,CD27,CD3D,CD3E,GIMAP1,GIMAP1-GIMAP5,HLA-DOA,HOPX,JAK3,Klrk1,LAT,LCK,LCP2,PSMB8,PTGS2,SASH3,TRPM2,UBD,Wfdc21,ZAP70 | 24 |
| Cell-To-Cell Signaling and Interaction | aggregation of cells | 5.99E-07 | Decreased | -2.34 | CCL5,CD2,CXCL9,ICAM3,LAT,LCK,LCP2,PTGS2,ZAP70 | 9 |
| Cellular Movement, Hematological System Development and Function, Immune Cell Trafficking | cell movement of leukocytes | 8.45E-07 | Decreased | -2.42 | CCL5,CCR5,CD2,CD3E,CXCL9,JAK3,LAT,LCK,LCP2,PRF1,PTGS2,TRPM2,ZAP70 | 13 |
| Free Radical Scavenging | synthesis of reactive oxygen species | 2.00E-06 | Decreased | -2.12 | CCL5,CCR5,CXCL9,LAT,LCK,LCP2,PRF1,PTGS2,TRPM2,ZAP70 | 10 |
| Cell-To-Cell Signaling and Interaction, Hematological System Development and Function, Immune Cell Trafficking | adhesion of mononuclear leukocytes | 2.39E-06 | Decreased | -2.43 | CCL5,CD2,CXCL9,JAK3,LCP2,ZAP70 | 6 |
| Developmental Disorder | Hypoplasia | 3.23E-06 | Increased | 3.08 | CD3E,GIMAP1-GIMAP5,JAK3,Klrk1,LAT,LCK,LCP2,PRF1,PTGS2,SASH3 | 10 |
| Cell-To-Cell Signaling and Interaction, Cell-mediated Immune Response, Cellular Movement, Hematological System Development and Function, Immune Cell Trafficking | adhesion of T lymphocytes | 4.66E-06 | Decreased | -2.21 | CD2,CXCL9,JAK3,LCP2,ZAP70 | 5 |
| Developmental Disorder, Immunological Disease, Organismal Injury and Abnormalities | hypoplasia of thymus gland | 7.31E-06 | Increased | 2.17 | CD3E,JAK3,LAT,LCK,PRF1 | 5 |
| Developmental Disorder | hypoplasia of organ | 8.08E-06 | Increased | 2.92 | CD3E,GIMAP1-GIMAP5,JAK3,Klrk1,LAT,LCK,LCP2,PRF1,PTGS2 | 9 |
| Cellular Movement | homing of cells | 7.61E-05 | Decreased | -2.35 | CCL5,CCR5,CXCL9,CXCR6,JAK3,LAT,LCK,PTGS2,TRPM2 | 9 |
| Cellular Growth and Proliferation | proliferation of cells | 1.98E-04 | Decreased | -2.42 | CARD11,CCL5,CCR5,CD2,CD27,CD3E,CD5,CXCL9,DNAJA1,GPR174,HOPX,ICAM3,JAK3,Klrk1,LAT,LCK,LCP2,PFKFB3,PRF1,PTGS2,RASSF4,SASH3,TRPM2,UBD,ZAP70 | 25 |
| Cellular Movement | cell movement | 6.20E-04 | Decreased | -2.36 | ABI3,ARHGEF28,CCL5,CCR5,CD2,CD3E,CXCL9,CXCR6,DNAJA1,JAK3,LAT,LCK,LCP2,PRF1,PTGS2,TRPM2,ZAP70 | 17 |
| Cellular Movement | migration of cells | 1.45E-03 | Decreased | -2.14 | ABI3,ARHGEF28,CCL5,CCR5,CD2,CD3E,CXCL9,JAK3,LAT,LCK,LCP2,PRF1,PTGS2,TRPM2,ZAP70 | 15 |
| Cellular Movement | chemotaxis | 1.64E-03 | Decreased | -2.13 | CCL5,CCR5,CXCL9,LAT,LCK,PTGS2,TRPM2 | 7 |

### Table 16 Diseases and functions in Violet module in Jersey cows

| Categories | Diseases or Functions Annotation | p-Value | Predicted Activation State | Activation z-score | Molecules | # Molecules |
| --- | --- | --- | --- | --- | --- | --- |
| Gastrointestinal Disease, Organismal Injury and Abnormalities | abnormality of large intestine | 3,53E-03 | Decreased | -2 | CBLB,FAAH,MERTK,MPC1,PLD1,PON1,TP53,ZNF784 | 8 |
| Gastrointestinal Disease, Inflammatory Disease, Inflammatory Response, Organismal Injury and Abnormalities | colitis | 7,08E-03 | Decreased | -2 | FAAH,MERTK,MPC1,PLD1,PON1,TP53,ZNF784 | 7 |
